# Programmable Release of Chemotherapeutics from Ferrocene‐Based Injectable Hydrogels Slows Melanoma Growth

**DOI:** 10.1002/adhm.202400265

**Published:** 2024-07-15

**Authors:** Rebecca Rothe, Yong Xu, Johanna Wodtke, Florian Brandt, Sebastian Meister, Markus Laube, Pier‐Luigi Lollini, Yixin Zhang, Jens Pietzsch, Sandra Hauser

**Affiliations:** ^1^ Helmholtz‐Zentrum Dresden‐Rossendorf Institute of Radiopharmaceutical Cancer Research Department of Radiopharmaceutical and Chemical Biology Bautzner Landstrasse 400 01328 Dresden Germany; ^2^ Technische Universität Dresden Faculty of Chemistry and Food Chemistry School of Science Bergstrasse 66 01069 Dresden Germany; ^3^ B CUBE Center for Molecular Bioengineering Technische Universität Dresden Tatzberg 41 01307 Dresden Germany; ^4^ Alma Mater Studiorum University of Bologna Department of Medical and Surgical Sciences Viale Filopanti 22 Bologna 40126 Italy

**Keywords:** biodegradable, local drug delivery, noncovalent hydrogel, sequential drug release, small animal imaging

## Abstract

Hydrogel‐based injectable drug delivery systems provide temporally and spatially controlled drug release with reduced adverse effects on healthy tissues. Therefore, they represent a promising therapeutic option for unresectable solid tumor entities. In this study, a peptide‐starPEG/hyaluronic acid‐based physical hydrogel is modified with ferrocene to provide a programmable drug release orchestrated by matrix‐drug interaction and local reactive oxygen species (ROS). The injectable ROS‐responsive hydrogel (hiROSponse) exhibits adequate biocompatibility and biodegradability, which are important for clinical applications. HiROSponse is loaded with the two cytostatic drugs (hiROSponse^dox/ptx^) doxorubicin (dox) and paclitaxel (ptx). Dox is a hydrophilic compound and its release is mainly controlled by Fickian diffusion, while the hydrophobic interactions between ptx and ferrocene can control its release and thus be regulated by the oxidation of ferrocene to the more hydrophilic state of ferrocenium. In a syngeneic malignant melanoma‐bearing mouse model, hiROSponse^dox/ptx^ slows tumor growth without causing adverse side effects and doubles the relative survival probability. Programmable release is further demonstrated in a tumor model with a low physiological ROS level, where dox release, low dose local irradiation, and the resulting ROS‐triggered ptx release lead to tumor growth inhibition and increased survival.

## Graphical abstract

An injectable peptide‐starPEG/hyaluronic acid hydrogel is modified with ferrocene (hiROSponse) to provide a programmable drug release. Loaded with doxorubicin and paclitaxel, hiROSponse^dox/ptx^ releases drugs in response to drug‐matrix interaction and local reactive oxygen species. When injected intratumorally, hiROSponse^dox/ptx^ slows tumor growth and thereby doubles survival probability in mouse models of malignant melanoma.

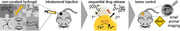

## 1 Introduction

Hydrogels are porous hydrophilic polymers used for a variety of biomedical applications such as tissue regeneration, wound healing, and cancer therapy. They can be tailored to possess inherent biocompatibility, favorable drug loading capacities, and controllable drug release features and can serve as efficient drug delivery systems with temporally and spatially defined release.^[^ ^1^ ^]^ Self‐assembled noncovalent hydrogels could provide injectability, tunable physical characteristics, and permeability to oxygen and other nutrients required for tissue regeneration. In hydrogel‐based treatment approaches of solid tumors, the hydrogels are injected locally near the tumor or directly into the tumor tissue in a minimally invasive manner to deliver the encapsulated therapeutic agents directly into the tumor tissue over a defined period of time.^[^ ^2^ ^]^ Spatially and temporally defined local release of therapeutics can reduce the undesirable side effects of conventional treatment approaches, in which therapeutics are often administered systemically, in high doses and in repeated cycles. Depending on the area of application or molecular characteristics of solid tumor entities, a programmable release in response to a stimulus can further enhance the spatial and temporal effect. Hydrogels can be tailored with a wide variability of modifiable mechanic and chemical properties, multifunctional modifications, controllable degradability, drug embedding techniques, and targeted stimulus‐dependent drug release kinetics.^[^ ^3^ ^]^ Incorporating hydrophobic drugs in hydrogels is relatively more difficult, but could be overcome, for example, by including cyclodextrins into the hydrogels.^[^ ^1a^ ^]^ In addition, the combined release of hydrophilic and hydrophobic therapeutics from a hydrogel often presents challenges in terms of designing a network with opposite chemical and physical features. In order to avoid the burst release of drugs and the resulting short‐term therapeutic effects, controlled release mechanisms can be used that exploit stimuli of the tumor microenvironment (TME), such as low pH, high redox potential, or overexpressed proteases.^[^ ^1^, ^4^ ^]^ In this study, both hydrophilic and hydrophobic drugs are incorporated in a stimuli‐responsive hydrogel based on ferrocene (hiROSponse^dox/ptx^), and the therapeutic effect of the programmable drug release was tested in murine malignant melanoma models.

Malignant melanoma, a melanocyte‐derived tumor, is one of the most aggressive skin malignancies with a steadily increasing incidence worldwide. The median survival of patients with highly metastasized, inoperable malignant melanoma (stage IV) is only 8 to 10 months and the 5‐year survival rate is approximately 10–20%.^[^ ^5^ ^]^ If malignant melanoma is detected at an early stage, it can be treated very well by surgical excision, whereas for advanced stage tumors efficient treatment options are lacking. In addition to surgical excision, standard treatment for malignant melanoma currently includes chemotherapy, radiotherapy, and immunotherapy.^[^ ^6^ ^]^ The systemic application of chemotherapy and immunotherapy is associated with sometimes severe side effects, such as renal toxicity, hepatitis, abdominal pain, loss of appetite, nausea, or fatigue.^[^ ^3^, ^5^, ^6^, ^7^ ^]^ Additionally, malignant melanocytes rapidly develop resistance toward therapeutic agents administered in the course of conventional treatments. Besides surgical excision, to date, only the intralesional treatment with immune modulatory agents or the combination therapy with cytostatic agents and electroporation are approved. Among these, the intratumoral application of a high dose of interleukin‐2 (IL‐2), with response rates exceeding 80% with repeated administration of the drug, especially in smaller metastases, is particularly recommended for the treatment of unresectable locoregional metastases of malignant melanoma.^[^ ^8^ ^]^ PV‐10, a 10% solution of rose bengal, is also suitable for intralesional administration and showed acceptable complete response rates (42%) of the injected lesions in an exemplarily chosen clinical trial.^[^ ^9^ ^]^ However, the to‐date limited therapeutic success highlights the need for the development and validation of new efficient melanoma treatment strategies.^[^ ^6^, ^10^ ^]^ Currently, hydrogel‐based drug delivery systems are commercially available only for local therapy of brain, prostate or breast tumors, but not for malignant melanoma.^[^ ^11^ ^]^

We have established a modular, noncovalently assembled hydrogel platform consisting of a repetitive lysine‐alanine peptide ((KA)_7_)‐starPEG conjugate and sulfated polysaccharides.^[^ ^12^ ^]^ These hydrogels are injectable and have favorable mechanical properties, such as viscoelasticity, appropriate self‐healing, and shear‐thinning behavior, guaranteeing a minimally invasive application. In this work, we have further developed the modular hydrogel to serve as a local drug delivery system by introducing a ferrocene (FeCp_2_) component within the hydrogel network (hiROSponse). FeCp_2_ is an organometallic ring system with a core‐positioned iron atom. It can be switched to a more hydrophilic state by oxidation (Fe^2+^ → Fe^3+^) enabling the release of embedded hydrophobic therapeutics in a reactive oxygen species (ROS) responsive manner.^[^ ^13^ ^]^ The TME of malignant melanoma is characterized by a high redox potential. In this context, hydrogen peroxide is the most abundant and stable ROS, with a higher concentration (50 × 10^−6^ m to 1 × 10^−3^ m) compared to healthy tissue (20 × 10^−9^ m). Besides the intrinsic ROS level, ROS can be generated by external irradiation as well as by Fenton reaction leading to a hydroxyl radical, a hydroxide ion, and an Fe^3+^‐containing product that contribute to apoptosis or ferroptosis of tumor cells.^[^ ^14^ ^]^ Further, the oxidation of FeCp_2_ by ROS within the hydrogel network is supposed to produce Fe^2+^ catalyzing the Fenton reaction.^[^ ^15^ ^]^ In hiROSponse, the cytostatic drugs doxorubicin (dox) and paclitaxel (ptx) were embedded (hiROSponse^dox/ptx^) to study the local drug release kinetics and tumor control effects as these therapeutic agents are the most widely used chemotherapeutic agents in clinical practice for the treatment of solid tumor entities.^[^ ^16^ ^]^ The redox‐dependent drug release by a FeCp_2_ component was recently shown for dox embedded in a FeCp_2_‐containing polycaprolactone‐based microgel and released by stimulation with hydrogen peroxide.^[^ ^17^ ^]^ The programmable release system consists of the sequential release of dox and ptx based on drug‐matrix interaction, a local low dose irradiation and the resulting ROS trigger to accelerate the release of ptx, and the potential effect of ferroptosis and Fenton reaction. The aim of this study was the development and characterization of hiROSponse for targeted local drug release to address malignant melanoma as a model tumor entity.

## 2 Results

### 2.1 Characteristics of hiROSponse

**Scheme 1:**
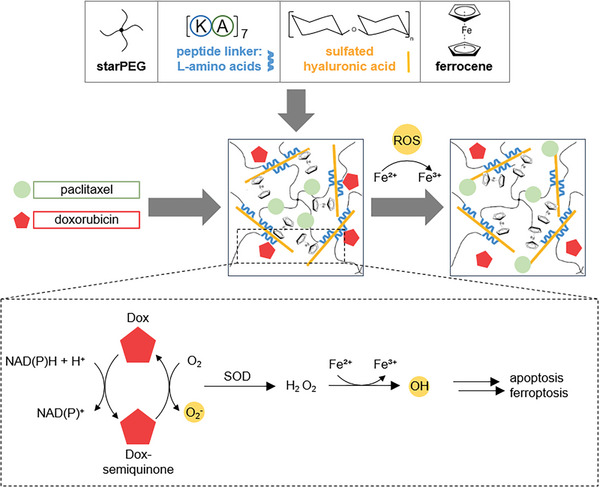
Modular building blocks of hiROSponse and reactive oxygen species (ROS) dependent drug release mechanism.

**Figure 1:**
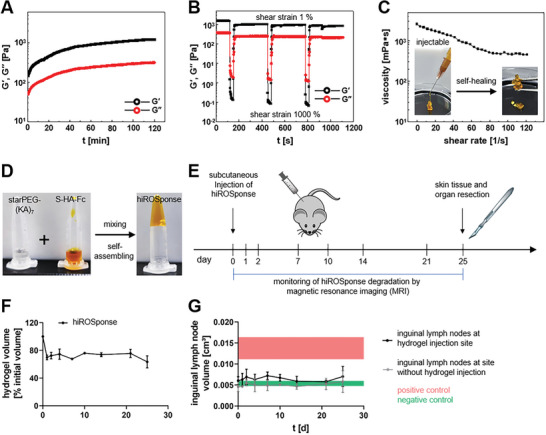
Characteristics of hiROSponse, in vivo degradation and biocompatibility in healthy SKH1 mice. A) Gelation of the self‐assembled bulk hydrogel hiROSponse. Self‐healing properties of hiROSponse with different stiffnesses. B) The corresponding recovery of hiROSponse undergoing cyclic deformation of 1% and 1000% strain at 1 Hz. C) Continuous flow experiments showing the viscosity and shear‐thinning behavior of hiROSponse. Insert images: hiROSponse can be extruded from a syringe through a 27G needle onto a plastic dish. D) Images illustrating the bulk gel formation of hiROSponse after mixing of starPEG‐(KA)_7_ and S‐HA‐FeCP_2_. E) Sequence of in vivo investigation on hiROSponse degradation and biocompatibility. F) Quantified hiROSponse volume (normalized to day 0) determined by magnetic resonance imaging (MRI) over an investigation period of 25 days, *n* = 3, mean ± SD. G) Quantified inguinal lymph node sizes at hydrogel injection site (black) and contralateral site without injected hydrogel (gray) determined by MRI, and in comparison to negative (untreated) and positive (TPA injection) controls shown in green and red, respectively, *n* = 3, mean ± SD.

**Scheme** **1** illustrates the noncovalent hydrogel system for the production of hiROSponse assembled by noncovalent interactions between a peptide and sulfated polysaccharides. The peptide, denoted as CWGG‐(KA)_7_ (KA_7_), was synthesized using standard Fmoc solid‐phase peptide synthesis and verified through liquid chromatography‐mass spectrometry (LC‐MS) (Figure S1A, Supporting Information). Subsequently, maleimide‐modified starPEG was linked to the peptide through Michael‐type addition. The successful synthesis of peptide‐starPEG conjugate was confirmed via NMR measurements (Figure S1B, Supporting Information). The FeCp_2_‐conjugated GAG (S‐HA‐FeCp_2_) was created by initially sulfating low molecular weight HA (130–300 kDa) (Figure S1C, Supporting Information). Sulfation was validated through attenuated total reflectance‐Fourier transform infrared (ATR‐FTIR) spectroscopy, which revealed a prominent peak corresponding to S═O stretching at 1230 cm^−1^ (Figure S1D, Supporting Information). To synthesize S‐HA‐FeCp_2_, ferrocenecarboxylic acid was coupled to S‐HA, and validated by ATR‐FTIR, where the peak associated with C─C stretches in the aromatic ring from 1500 to 1400 cm^−1^ was observed (Figure S1D, Supporting Information). This self‐assembling system was employed for the synthesis of bulk hiROSponse by mixing peptide‐starPEG and S‐HA‐FeCp_2_ (**Figure** **1**D), with validation performed via rheology tests to monitor the gelation process (Figure 1A). Notably, the noncovalent network of hiROSponse exhibited shear‐thinning behavior (Figure 1B) and has shown self‐healing capabilities (Figure 1C), two essential rheological properties for injectability and printability. HiROSponse could be smoothly extruded from a syringe using a 27G needle as a stable material (Figure 1C and Movie S1, Supporting Information). Moreover, hiROSponse remained injectable even after 24 h, while being usable for injection shortly (30 min) after the mixing of the two precursors.

### 2.2 hiROSponse is Slowly Degrading and Biocompatible in Immunocompetent Mice

The in vivo degradability and biocompatibility of hiROSponse were investigated to demonstrate its applicability as drug release system that should be stable during a short‐term treatment of a few weeks without eliciting adverse tissue reactions. An amount of 50 µL of hiROSponse were subcutaneously injected in immunocompetent hairless Crl:SKH1‐Hrhr mice. The hydrogel degradation was investigated over a period of 25 days by small animal magnetic resonance imaging (MRI) using a T2‐weighted measuring sequence (Figure 1E and Figure S2A, Supporting Information). Shortly after injection, an accumulation of tissue fluid around hiROSponse was observed, which subsided within 1 day (Figure S2A, Supporting Information). The temporary fluid accumulation was accompanied with a hydrogel volume reduction of about 30 % within the first 24 h after hiROSponse injection, probably in part due to hydrolytic cleavage of the hydrogel network (Figure 1F). Hereinafter, the hydrogel volume remained stable over the study period of 1 month.

The size of inguinal lymph nodes was investigated using the same MRI measurement setting concurrently (Figure S2A, Supporting Information) as swollen lymph nodes could be a first sign of an inflammatory reaction to hiROSponse injection. The size of the inguinal lymph nodes was analyzed by comparing with TPA (12‐O‐tetradecanoylphorbol‐13‐acetate) injected animals and untreated animals as positive and negative control, respectively.^[^ ^18^ ^]^ During the whole investigation period, the size of the lymph nodes was in the range of the negative control (Figure 1G).

HiROSponse biocompatibility regarding tissue morphology of excreting organs, such as liver, kidney, spleen and lymph nodes, and local reactions at the hydrogel‐tissue interface were determined ex vivo. In H&E overview stainings, neither in the hydrogel surrounding nor in the organ samples adverse structural changes of the tissue architecture were obvious (Figures S2B and S3A, Supporting Information). The thickness of the subcutaneous tissue layers around hiROSponse (247.39 ± 65.52 µm) determined by van Gieson's stain was comparable to the negative control (244.89 ± 46.00 µm), thus a detrimental fibrous hydrogel encapsulation could be excluded. Local tissue response towards hiROSponse was studied by immunohistochemical stainings of several inflammation markers (cyclooxygenase‐2 (COX‐2), thrombomodulin (TM) and receptor of advanced glycation end products (RAGE)), infiltration of pan‐macrophages (CD68), angiogenesis (VEGF) and blood vessel formation (CD31), matrix remodeling (TG‐2) as well as proliferation marker (Ki67). In the hydrogel surroundings, no enhanced antigen expression of the analyzed marker proteins could be detected as the quantifications of stained areas were in the range of the negative control (Figure S3B and Table S1, Supporting Information). Basal proliferative activity and matrix remodeling were visible ex vivo in the epidermis. In accordance with the overview staining for the assessment of skin and organ tissue constitution as well as the analysis of the inguinal lymph node size in vivo, hiROSponse can be regarded as quasi‐inert.

Since hiROSponse did not induce adverse host tissue reactions and showed a favorable short‐term stability, it was applied in drug delivery studies using a syngeneic melanoma‐bearing C57BL/6JRj mouse model.

### 2.3 hiROSponse Degrades and Slows Tumor Growth in a Mouse Melanoma Model

**Figure 2:**
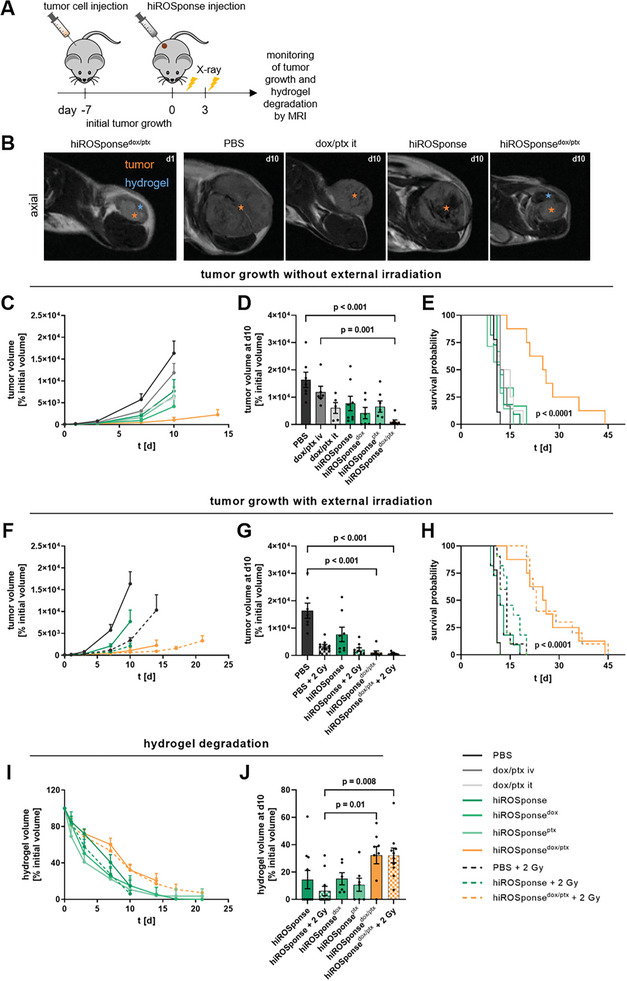
HiROSponse^dox/ptx^ slows tumor growth in B16F10 melanoma‐bearing C57BL/6JRj mice. A) Schematic experimental procedure. B) Representative magnetic resonance imaging (MRI) of subcutaneous solid B16F10 tumor (orange mark) and injected hydrogel (blue mark) at d1 and d10 after hiROSponse injection. C–E) Monitoring of tumor growth without external irradiation. F–H) Monitoring of tumor growth with external irradiation. C,F) B16F10 tumor volumes (normalized to the initial volume) determined by MRI, *n* = 6–11 at d0, mean + SEM. D,G) B16F10 tumor volumes at d10, *n* = 5–10, mean ± SEM, one‐way ANOVA, Bonferroni post‐hoc test. E,H) Calculated relative survival probability depicted as Kaplan–Meier plot, *n* = 6–11, Log‐rank test. I) Hydrogel volumes (normalized to the initial volume) determined by MRI, *n* = 8–11 at d0, mean + SEM. J) Hydrogel volumes at d10; *n* = 6–11, mean ± SEM, one‐way ANOVA, Bonferroni post‐hoc test.

For tumor control studies, murine B16F10 melanoma cells were subcutaneously injected in C57BL/6JRj mice creating a syngeneic mouse melanoma model. After an initial tumor growth period of 7 days, leading to tumors of approx. 100 mm^3^, the respective hiROSponse was injected intratumorally (**Figure** **2**A). Tumor growth and hydrogel degradation were monitored using MRI (Figure 2B). This study design would correspond to a clinical situation of malignant melanoma patients with local lesions that are treated with local hydrogel injection.

In comparison to the control treatment groups, including local injection of PBS, dox/ptx, hiROSponse, hiROSponse^dox^ or hiROSponse^ptx^, hiROSponse^dox/ptx^ slowed tumor growth significantly (Figure 2C,D). The different tumor growth profiles are reflected by the calculated descriptive parameters, such as doubling time and tumor growth rate (Figure S4A, Table S2, Supporting Information). The highest doubling time was determined for the hiROSponse^dox/ptx^ group with 3.34 ± 1.33 d and was 1.4‐times higher compared to the local injection of dox/ptx and 1.8‐times higher compared to the local injection of PBS. Consequently, the hiROSponse^dox/ptx^ group exhibited the lowest tumor growth rate constant with 0.21 ± 0.08 d^−1^. Based on an exponential growth model, relative survival probabilities were calculated (Figure 2E). hiROSponse^dox/ptx^ significantly increased the survival of the tumor‐bearing mice to a maximum of 45 days, thereby more than doubling the relative survival probability compared to all control treatment groups.

In addition, we have examined whether local external irradiation of the tumor area could influence hydrogel‐based drug release and thus tumor growth (Figure 2F,G). A low dose of 2 Gy was chosen in order not to induce significant therapeutic effects solely by the applied external irradiation. Local irradiation was intended to further increase the ROS level within the TME and thus trigger the ptx release upon reducing the network hydrophobicity. External irradiation with 2 Gy slightly slowed tumor growth in the control (PBS + 2 Gy) and hydrogel groups (hiROSponse + 2 Gy; hiROSponse^dox/ptx^ + 2 Gy) compared to the respective nonirradiated groups (PBS; hiROSponse; hiROSponse^dox/ptx^). This trend was also evident in terms of the relative survival probability, however, without showing major synergistic effects (Figure 2H). We additionally performed experiments with external irradiation of 5 Gy after hiROSponse^dox/ptx^ application to artificially enhance the ROS levels even more. This did not result in a significantly different effect on tumor volume as compared to irradiation with 2 Gy (Figure S4B, Supporting Information).

Degradation of the hydrogels was accelerated by rapid tumor growth leading to negative correlations between tumor and hydrogel volumes for all hiROSponse‐injected groups (Figure S5, Supporting Information). Accordingly, hiROSponse, hiROSponse^dox^, and hiROSponse^ptx^ showed significantly faster degradation profiles compared to hiROSponse^dox/ptx^ (Figures 2I,J). Ten days after hydrogel injection, >30% of the initial hydrogel volume of hiROSponse^dox/ptx^ was still detectable, whereas the volumes of hiROSponse, hiROSponse^dox^, and hiROSponse^ptx^ were reduced to <15%. The external irradiation did not result in differences in hydrogel degradation compared to the respective nonirradiated groups. The different degradation profiles of the hydrogels could be calculated as hydrogel degradation rates. The slopes of hiROSponse degradation (−8.22 ± 0.65 %V d^−1^, without irradiation; and −9.17 ± 0.72 %V d^−1^, with irradiation) were 1.5‐ to 2.1‐fold steeper compared to hiROSponse^dox/ptx^ without irradiation (−5.48 ± 0.46 %V d^−1^) and with irradiation (−4.36 ± 0.29 %V d^−1^) (Table S2, Supporting Information).

In conclusion, the local release of dox and ptx from hiROSponse^dox/ptx^ delayed tumor growth and significantly increased the relative survival probability.

### 2.4 Hydrophilic and Hydrophobic Drug are Released Sequentially from hiROSponse^dox/ptx^ Triggered by Tumor Intrinsic ROS

In the design of hiROSponse^dox/ptx^, we aimed at a sequential drug release. As a relatively hydrophilic drug with a logP value of 0.53, dox should be rapidly released from the hydrogel by simple diffusion. In contrast, ptx with a higher logP value of 3.54 was embedded in the hydrogel network and can interact with FeCp_2_ through hydrophobic interaction. ROS‐induced oxidation of FeCp_2_, switching the oxidation state from Fe^2+^ to Fe^3+^ and thus the hydrophobicity in a suspected redox cycling process,^[^ ^19^ ^]^ was thought to trigger the local release of ptx into the TME. In vitro, release of dox was shown to be faster than ptx release, which was even more pronounced in the presence of ROS (Figure S6A, Supporting Information). Further, the hypothesized changes in hydrophilicity induced by the presence of ROS were confirmed by contact angle measurements (Figure S6B, Supporting Information). To study the release kinetics of dox and ptx in vivo, peripheral blood samples from mice of the tumor control studies were collected and the concentrations of intact dox and ptx in pooled plasma samples were analyzed by ultra‐high‐performance liquid chromatography coupled with tandem mass spectrometry (UPLC‐MS/MS). By that, the intact cytostatic agents, which enter the blood could be quantified and the release of dox and ptx could be followed. Of note, metabolic products of dox and ptx were not detected with the applied method.

**Figure 3:**
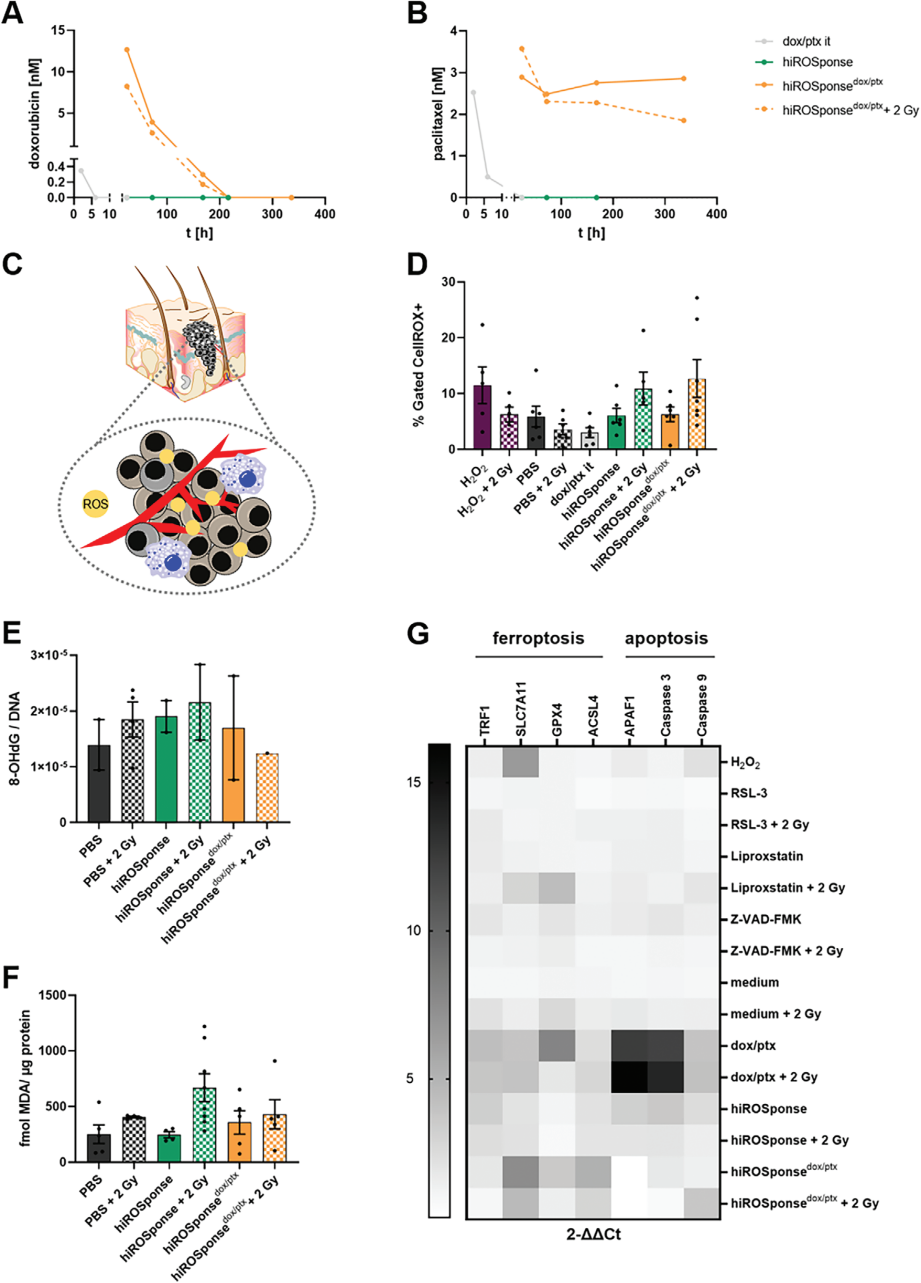
Drug release kinetics, local reactive oxygen species (ROS) levels and ferroptosis. A) Concentration of doxorubicin and B) paclitaxel in blood samples of B16F10 melanoma‐bearing C57BL/6JRj mice measured by ultra‐high‐performance liquid chromatography coupled with tandem mass spectrometry (UPLC‐MS/MS) (pooled samples of three animals per group). C) Scheme of intrinsic ROS level within the local tumor microenvironment (TME). D) Determination of ROS level in B16F10 tumor samples using flow cytometry by gating cells according to a positive fluorescence signal of the CellROX probe (H_2_O_2_: 50 µL of 0.1% solution it); *n* = 4–7, mean ± SEM. E) 8‐OHdG (8‐hydroxy‐2′‐deoxyguanosine) assay of B16F10 tumor samples 3 days after hydrogel injection (8‐OHdG concentration normalized to DNA concentration), *n* = 1–4, mean ± SEM. F) TBARS (thiobarbituric acid reactive substances) assay of B16F10 tumor samples 3 days after hydrogel injection, *n* = 4–8, mean ± SEM. G) Relative mRNA expression of specific genes for ferroptosis (TRF1 – transferrin receptor 1, SLC7A11 – sodium‐independent cystine‐glutamate antiporter, GPX4 – glutathione peroxidase 4, ACSL4 – long‐chain‐fatty‐acid‐CoA ligase 4) and apoptosis (APAF1 – apoptotic protease‐activating factor 1). Control group (medium) served as reference and was set to 2^−ΔΔCt^ = 1, *n* = 2–3 (8 spheroids pooled for each experiment).

In the hiROSponse^dox/ptx^ and the hiROSponse^dox/ptx^ + 2 Gy groups, dox concentration dropped from almost 10 × 10 ^−9^ m to less than 0.4 × 10 ^−9^ m within 3 days, reflecting a rapid release (**Figure** **3**A). In the same hydrogel groups, a long‐lasting ptx concentration of 2 × 10 ^−9^ to 3 × 10 ^−9^ m was observed indicating a sustained release over at least 1 week (Figure 3B). In comparison, intratumorally injected dox/ptx was rapidly cleared from the body as neither dox nor ptx were detectable in the murine plasma as early as 5 h after injection. Thus, hiROSponse^dox/ptx^ functioned as sequential drug depot with short‐term release of dox and long‐lasting ptx release. This interplay locally exerted both immediate and sustained effects on tumor cells, and significantly delayed the overall tumor growth (Figure 2).

A high local ROS level is needed for the oxidation of FeCp_2_. The TME of malignant melanoma shows a mild acidic pH of 5.5 to 6.5.^[^ ^20^ ^]^ and thus does not provide the optimal pH for Fenton reaction, which requires an acidic pH of 2 to 4.^[^ ^21^ ^]^ As the local ROS level of the TME was intended to trigger the ptx release (Figure 3C), intrinsic ROS level of the malignant melanoma model and the additionally generated ROS by external irradiation were quantified. Since ROS quantification by intraperitoneal injection of luminol derivative L‐012 and in vivo chemiluminescence imaging resulted in large variations between individual mice and measurement days (see Experimental Section in Supporting Information, Figure S7A, Supporting Information), ROS were quantified ex vivo in resected tumor samples using the fluorescent CellROX probe and analyzed by flow cytometry (Figure 3D). ROS were detected to be of a similar extent in both the control groups (PBS and dox/ptx) and the hydrogel groups (hiROSponse; hiROSponse^dox/ptx^) and were found to be comparable to the positive control (with H_2_O_2_ injection). The ROS level was slightly increased by external irradiation with 2 Gy for both hiROSponse and hiROSponse^dox/ptx^.

According to literature, tumors expressing mesenchymal markers rather than epithelial marker proteins are more sensitive to ferroptosis.^[^ ^22^ ^]^ Mesenchymal tumors have increased levels of polyunsaturated fatty acid‐containing phospholipids (PUFA‐PL) driving the cells towards glutathione peroxidase 4 (GPX4) dependency. Moreover, mesenchymal tumor cells highly express CD44 mediating HA‐dependent iron endocytosis. In the malignant melanoma model used in this study, high local ROS of the TME in combination with the FeCp_2_‐containing hydrogel system may induce ferroptosis, which could have further contributed to some extent to tumor cell death. Therefore, immunohistochemical staining of several epithelial markers (cytokeratin‐19 (KRT19), epithelial cell adhesion molecule (EpCAM), laminin, and occludin) and mesenchymal markers (HA receptor CD44, fibronectin, and alpha smooth muscle actin (α‐SMA)) in sectioned B16F10 tumor tissues were performed to determine whether the chosen model tumor entity is sensitive to ferroptotic processes (Figure S7B, Supporting Information). Although melanocytes are of neuroectodermal origin,^[^ ^5d^ ^]^ B16F10 tumors primarily express mesenchymal markers, especially CD44 and α‐SMA, making them particularly sensitive to ferroptosis. Epithelial markers (KRT19 and laminin) were not or only detectable as a nonspecific weak background staining (EpCAM; occludin).

As B16F10 tumors are probably sensitive to ferroptosis, iron‐containing hiROSponse^dox/ptx^ could have elicited additional ferroptotic processes in the tumors. These can result in the formation of 8‐hydroxy‐2′‐deoxyguanosine (8‐OHdG) and, via lipid peroxidation, of malondialdehyde (MDA) or 4‐hydroxynoneal (4‐HNE) reflecting cellular oxidative stress (Figure S8A, Supporting Information). Therefore, extracted tumor samples were investigated regarding DNA damage (8‐OHdG) and lipid peroxidation products. Both 8‐OHdG and MDA products were detected in the tumor samples (Figure 3E,F) in comparable amounts in the hiROSponse groups and the PBS group. In irradiated tumor samples, MDA formation increased by 1.2‐ to 2.7‐fold (fmol MDA per µg protein) compared to the corresponding nonirradiated samples. Further, the similar presence of lipid peroxidation products was confirmed by immunohistochemical staining of 4‐HNE in sectioned tumor samples of PBS and hydrogel‐treated animals (Figure S8B, Supporting Information). Additionally, qPCR experiments were performed using B16F10 spheroids to investigate the expression of ferroptosis‐ and apoptosis‐associated genes (Figure 3G). A two‐fold change of expression was calculated for hiROSponse and hiROSponse + 2 Gy in terms of TRF1 (transferrin receptor 1), SLC7A11 (sodium‐independent cystine‐glutamate antiporter), and ACSL4 (long‐chain‐fatty‐acid‐CoA ligase 4), whereas hiROSponse^dox/ptx^ resulted in a fold change expression of >3 for SLC7A11, GPX4, and ACSL4. Additional effects of the external irradiation were not detected. Soluble dox/ptx increased the expression (2^−ΔΔCt^ >2) in all investigated ferroptosis genes. In contrast, only an altered expression in SLC7A11 was visible in H_2_O_2_ treated spheroids. While hiROSponse^dox/ptx^ showed major effects on ferroptosis‐associated genes, hiROSponse led to a change of expression >3‐fold regarding the apoptosis‐associated genes APAF1 (apoptotic protease‐activating factor 1), caspase 3, and caspase 9. Major alterations in gene expression were also apparent in dox/ptx treated spheroids, with no additional effects by external irradiation. Overall, soluble dox/ptx upregulated apoptosis‐related gene expression, while hiROSponse^dox/ptx^, but not hiROSponse, induced ferroptosis‐related genes. These effects were independent of additional external irradiation with 2 Gy. However, in treated tumors ferroptosis products, such as 8‐OHdG or MDA, were not changed by treatment with hiROSponse^dox/ptx^ compared to hiROSponse or PBS.

### 2.5 hiROSponse^dox/ptx^ does not Induce Adverse Systemic Side Effects

With regard to potential clinical applications, systemic adverse effects must be investigated and excluded in the disease model. During the experiments, the mice were examined for the following possible side effects: enlarged inguinal lymph nodes, increased white blood cell count (WBC) in peripheral blood samples, increased protein excretion in spontaneous urine samples, morphological tissue changes in resected liver and kidney samples, and changes in the weight of the mice over the experimental period. The size of the inguinal lymph nodes was examined by MRI (Figure S9, Supporting Information). The inguinal lymph node volumes at the tumor‐bearing site slightly increased with no significant differences among the experimental groups. Tumor growth itself is associated with inflammatory reactions being reflected by the lymph node volume increase.^[^ ^23^ ^]^ The inguinal lymph nodes at the contralateral site without a B16F10 tumor remained small in size during the whole experimental period suggesting the absence of excessive systemic inflammation. The WBC of the hiROSponse^dox/ptx^‐treated mice was in the range or even lower than that of the untreated melanoma‐bearing mice (Figure S10A, Supporting Information), excluding dysregulated immune reactions. To assess kidney function and possible functional disorders, the protein concentration in the urine was measured. Compared to the negative control, the protein concentration in collected mouse urine samples increased with increasing tumor growth (Figure S10B, Supporting Information). Likewise, the pH value of the urine samples of the treatment groups was slightly more acidic compared to the negative control (Figure S11, Supporting Information). These values might indicate a renal abnormality so that renal dysfunction and proteinuria was assessed via the total protein to creatinine ratio. In all experimental groups, this ratio was lower than the negative control (Figure S10C, Supporting Information). The protein concentration ratios in urine samples tended to increase with increasing duration of the experiment, first for hiROSponse and then for hiROSponse^dox/ptx^. The excretory organs were examined histologically and no pathological changes were apparent in either liver or kidney samples (Figure S10D, Supporting Information). The weight development was similar in all study groups (Figure S12, Supporting Information).

### 2.6 Melanin Contributes to Tumor Intrinsic ROS and Controls hiROSponse^dox/ptx^ Efficacy

In melanotic malignant melanoma cells, such as B16F10, redox reactions are necessary for the synthesis of melanin, but simultaneously lead to the formation of superoxide anion radicals or hydrogen peroxide, among others. Therefore, a basal intrinsic level of ROS and oxidative stress is already present in the TME of these tumors.^[^ ^24^ ^]^ In addition, the melanin pigment itself might have further influenced the therapeutic success of hiROSponse^dox/ptx^. Melanin can both accept and donate electrons and thus the present redox state of the pigment determines its contribution to the release mechanism from hiROSponse^dox/ptx^.^[^ ^25^ ^]^ To study the impact of the redox active melanin on the drug release mechanism and concomitantly tumor control effects, hiROSponse^dox/ptx^ was tested in a murine amelanotic B78H1 model.

**Figure 4:**
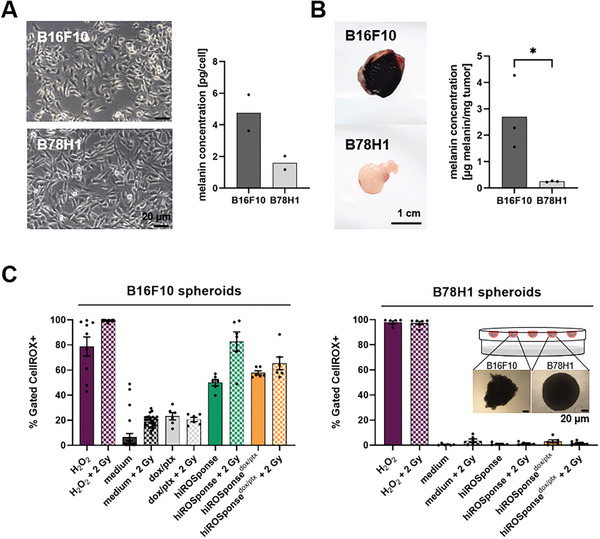
Melanin concentration and reactive oxygen species (ROS) levels in B16F10 and B78H1 melanoma. A) Cell morphology and melanin concentration of melanotic B16F10 and amelanotic B78H1 cells in monolayer cell cultures. B) Representative B16F10 and B78H1 tumors and melanin concentration in resected tumor samples, *n* = 3, two‐tailed unpaired *t*‐test, ^*^ *p* < 0.05. C) ROS level in B16F10 and B78H1 spheroids determined by flow cytometry (gating cells according to a positive fluorescence signal of the CellROX probe), *n* = 5–29, mean ± SEM. Inserted image shows representative microscopic image of B16F10 and B78H1 spheroids after 4 days of cultivation as hanging drops.

The amelanotic B78H1 melanoma cells, derived from an amelanotic B16F10 clone (see cell line authentication in Table S3, Supporting Information), have a similar cellular shape like melanotic B16F10 melanoma cells (**Figure** **4**A), but the tumor growth in vivo is slower leading to smaller and more compact tumors (Figure 4B). Determination of the melanin content in both monolayer and tumor samples confirmed the absence of melanin in amelanotic B78H1 samples in comparison to B16F10 samples (Figure 4A,B). To investigate the influence of melanin on ROS‐dependent drug release, the ROS levels of both models were compared. Due to the compact structure of the B78H1 tumors, these samples had to be mechanically minced prior to flow cytometric analysis with the CellROX probe. The mechanical processing presumably destroyed many B78H1 tumor cells resulting in the release of oxidative cellular substances. As a result, irregular ROS levels were measured (Figure S13C, Supporting Information). For this reason, the CellROX examinations were carried out with spheroid samples of both melanoma cell lines (Figure 4C). In B16F10 spheroids, an intrinsic ROS level was measurable in controls (PBS, dox/ptx) and hydrogel (hiROSponse and hiROSponse^dox/ptx^)‐treated samples, with H_2_O_2_‐treated sample as positive control. Additional external irradiation with 2 Gy increased the ROS level in comparison to the corresponding nonirradiated samples. In contrast, ROS was detectable in B78H1 spheroids only in the H_2_O_2_‐treated control reflecting an absent intrinsic ROS level.

**Figure 5:**
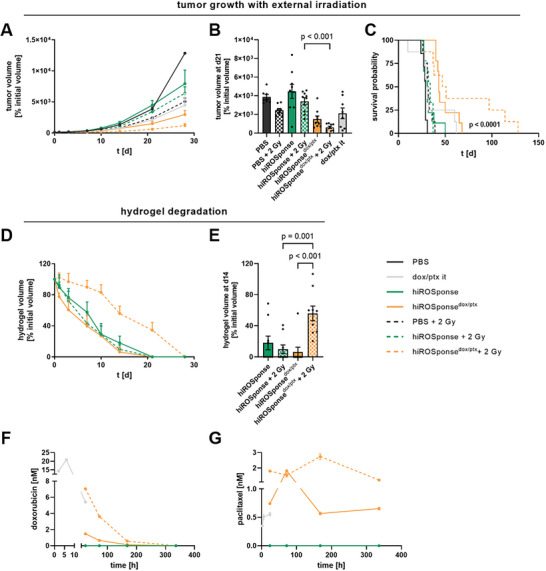
In vivo tumor control by hiROSponse^dox/ptx^ in amelanotic B78H1 melanoma‐bearing C57BL/6JRj mice. A) Quantified tumor volumes normalized to the initially injected hydrogel volumes (d0); *n* = 7–9, mean + SEM. B) Tumor volumes at d21; *n* = 7–9, mean ± SEM, one‐way ANOVA, Bonferroni post‐hoc test. C) Relative survival probability based on the 100‐fold increase in initial tumor volume using an exponential growth model; *n* = 7–9, Log‐rank test. D) Quantified hydrogel volumes normalized to the initially injected hydrogel volumes (d0); *n* = 7–9, mean + SEM. E) Hydrogel volumes at d14; mean ± SEM, one‐way ANOVA, Bonferroni post‐hoc test. F) Concentration of paclitaxel and G) doxorubicin in blood samples measured by mass spectrometry (pooled samples of three animals per group).

As intrinsic ROS could not be detected in the B78H1 cells in vitro, it was expected that no ROS‐dependent drug release from hiROSponse^dox/ptx^ in vivo would occur. We investigated whether an external irradiation can provide an additional gain‐of‐function mechanism, to create a ROS‐enriched environment as well as ROS‐induced drug release. B78H1 tumor growth in the hiROSponse^dox/ptx^ (+2 Gy) group was the slowest (**Figures** **5**A,B and S14, Supporting Information). According to the Kaplan–Meier curves, a doubling of relative survival probability was evident only in the hiROSponse^dox/ptx^ + 2 Gy group (Figure 5C). Remarkable, quantifying tumor growth, hiROSponse^dox/ptx^ + 2 Gy resulted in a 3‐ to 4‐fold increased doubling time, with about 16 ± 6 d, compared to the other groups, with doubling times ranging from 4 ± 1 to 6 ± 2 d (Table S4, Supporting Information). In contrast to the B16F10 model, hiROSponse^dox/ptx^ alone was not sufficient to double the relative survival probability. hiROSponse^dox/ptx^ + 2 Gy showed a delayed hydrogel degradation compared to all other experimental groups (Figures 5D,E and S15, Supporting Information), correlating with an inhibited tumor growth. Similar to the B16F10 model, adverse side effects could be excluded according to inguinal lymph node measurements and mouse weight (Figures S16 and S17, Supporting Information). A rapid release of dox from hiROSponse^dox/ptx^ and hiROSponse^dox/ptx^ + 2 Gy was evident, which was similar to the B16F10 model (Figure 5F). In the hiROSponse^dox/ptx^ group, because of the low local ROS, less than 1 × 10 ^−9^ m of ptx was measured by UPLC‐MS/MS after 2 h. In accordance with the tumor control effects of hiROSponse^dox/ptx^ + 2 Gy, the ptx plasma concentration was comparable to the B16F10 model with about 2 × 10 ^−9^ m over the course of investigation (Figure 5G). Intratumorally injected dox/ptx was detectable in the plasma samples only initially, being rapidly excreted and thus not delaying tumor growth in the long term.

## 3 Discussion

In this study, a noncovalently assembled hydrogel system was developed to serve as a targeted drug delivery system for local tumor therapy. HiROSponse^dox/ptx^ comprised of (KA)_7_ peptide‐starPEG conjugate, FeCp_2_‐modified S‐HA, and two incorporated cytostatic agents (dox and ptx) for programmable drug release. HiROSponse^dox/ptx^ was characterized regarding the general clinical requirements such as adequate physical stability and injectability, biocompatibility, biodegradability, and targeted drug release in malignant melanoma as model tumor entity.

The degradation of hiROSponse in healthy immunocompetent SKH1 mice was slower as compared to the degradation of (KA)_7_‐starPEG/S‐HA hydrogel of a previous study.^[^ ^12^ ^]^ showing that the additionally introduced FeCp_2_ component has altered the degradation properties in vivo. HiROSponse is biocompatible and proved to be quasi‐inert, which is in accordance with the literature describing the in vivo biocompatibility of FeCp_2_‐containing systems.^[^ ^26^ ^]^

Biodegradability of drug‐containing hiROSponse^dox/ptx^ in response to the physiological factors of the TME in a melanoma‐bearing immunocompetent C57BL/6J mouse model was investigated in advance of efficacy studies. HiROSponse^dox/ptx^ degraded faster in the melanoma‐bearing mouse model compared to the SKH1 mouse model. This was to be expected, as an increased expression of proteolytic enzymes, such as several MMPs and hyaluronidase, is characteristic of the invasive, metastatic nature of melanoma and promotes tumor progression.^[^ ^27^ ^]^ Further, significant differences were evident between hiROSponse^dox/ptx^ and the other hydrogel groups studied, which were due to the varying effects on the tumor growth. A significant delay in tumor growth and statistically relevant increase in relative survival of B16F10 melanoma‐bearing mice were achieved with hiROSponse^dox/ptx^ (+ 2 Gy). The doubling of the survival probability was accompanied by slower in vivo degradation of the hydrogels, presumably because of a lower proteolytic activity in the TME. Similar tumor control effects are described in the literature dealing with other drug delivery system compositions. For instance, a dextran‐based injectable hydrogel showed a degradation‐dependent release of 4 mg kg^−1^ dox and caused a significant reduction of tumor growth by approximately 50 % using a murine melanoma model, compared to the local injection of dox without hydrogel matrix.^[^ ^28^ ^]^ In addition to this monotherapy approach, combined cytostatic release from hydrogel systems had also been analyzed with regard to anti‐tumor efficacy. In a PEG‐based nanoparticle system, both hydrophilic dox (10 mg kg^−1^) and hydrophobic ptx (20 mg kg^−1^) were embedded in the core and shell regions of the particles, respectively. In vitro studies supported the anti‐tumor effects, suggesting a synergistic interaction of both released cytostatic drugs at a 2:1 ratio of dox to ptx.^[^ ^16^ ^]^ Further in a xenograft model, the combined release of dox and ptx from the respective PEG‐based nanoparticles, being embedded in a hybrid hydrogel system, resulted in a significant delay of tumor growth.^[^ ^29^ ^]^ In comparison to the mentioned studies, hiROSponse^dox/ptx^ elicited significant tumor control effects with both cytostatic agents being incorporated in a rather low dose of 5 mg kg^−1^ and ROS‐triggered release of the drugs within the TME.

The significant and prolonged tumor control effects of hiROSponse^dox/ptx^ indicated that the hydrogel acted as a local depot delivering the cytostatic agents continuously over time. In order to analyze the drug release kinetics in more detail, the concentration of dox and ptx in blood plasma samples, taken at different time points after hydrogel injection was determined. Dox and ptx were released sequentially, with a more rapid release of dox within 1 week and a sustained release of ptx over more than 2 weeks. A study using co‐delivery of 5 mg kg^−1^ dox and an immunotherapeutic agent by an injectable PEG‐based hydrogel system had showed significant anti‐tumoral effects in a B16F10 mouse model, but dox was not detectable in collected blood samples at d1 and d14 after hydrogel injection.^[^ ^30^ ^]^ In contrast, according to a reported elimination half‐life of approx. 5 h, the determined ptx concentrations were within the expected range.^[^ ^31^ ^]^ Moreover, applying 5 mg kg^−1^ ptx intravenously, other researchers have found concentrations of 0.15–1.25 × 10^−6^ m in rat plasma after 2 h, further decreasing to 15 × 10 ^−9^ m within 10 h. When applying ptx intratumorally, a slower release into the peripheral blood is expected due to a simultaneous distribution in the tumor and other nearby tissues, wherefore a lower plasma concentration can be assumed.^[^ ^32^ ^]^ In a different study co‐delivery of dox and ptx from a glycol‐chitosan‐based hydrogel significantly increased survival of B16F10 melanoma‐bearing mice compared to single drug release. Presumably, in this context, the burst release of dox contributed to a rapid inhibition of tumor growth, whereas the continuous ptx release was able to maintain this tumor‐therapeutic effect over a longer period of time. Hydrogel‐based ptx monotherapy exhibited smaller effects in terms of tumor growth and survival compared to dox‐based therapeutic approaches, again highlighting the particular efficacy of initial dox release.^[^ ^16^, ^33^ ^]^ This principle presumably also underlies the efficacy of hiROSponse^dox/ptx^ combining immediate effects mediated by dox and enduring effects due to the sustained ptx release. This enables the use of rather low drug concentrations that do not induce adverse systemic side effects, as confirmed by investigating inguinal lymph nodes, WBC, urine samples, mouse weight, and the morphology of excreting organs.

Local external irradiation of hiROSponse^dox/ptx^ with a low dose of 2 Gy did not enhance the effects on tumor growth or survival probability compared to nonirradiated hiROSponse^dox/ptx^. Irradiation with 2 × 2 Gy is below an efficient dose of 15 Gy, according to Smilowitz and colleagues,^[^ ^25^ ^]^ but can increase the local ROS level in tumor samples. However, release kinetics of dox and ptx were similar in irradiated and nonirradiated hiROSponse^dox/ptx^ groups. This confirms that sufficient ROS for adequate drug release was already present in the TME of B16F10 melanoma without external irradiation. These ROS are necessary for melanin synthesis, in which superoxide anion radicals or hydrogen peroxide are formed, among others.^[^ ^24^ ^]^ Moreover, the FeCp_2_ component of the hydrogel network is capable of generating hydrogen peroxide or reacting with hydrogen peroxide to produce hydroxyl radicals (Fenton reaction) by one electron oxidation, thus further increasing the local ROS level and inducing ferroptosis, a form of cell death.^[^ ^34^ ^]^ Besides the melanin pigment itself, glutathione and other redox‐active cofactors of the TME might also contribute to redox cycling of FeCp_2_.^[^ ^35^ ^]^ Although the analyses of 4‐HNE, 8‐OHdG, and MDA did not indicate upregulated ferroptotic processes in response to hiROSponse^dox/ptx^, qPCR investigations showed increased expression of ferroptosis‐associated genes.

As melanin pigments might have an influence on the ROS‐dependent release mechanism, the efficacy of hiROSponse^dox/ptx^ as well as the drug release kinetics were tested in an amelanotic murine malignant melanoma model having a very low intrinsic ROS level. In this B78H1 melanoma‐bearing model, local low‐dose irradiation of hiROSponse^dox/ptx^ was essential to significantly delay tumor growth and increase relative survival. Also, the tumor control was accompanied by a significantly delayed hydrogel degradation and higher drug concentrations detectable in plasma over a longer period of time. Without an intrinsic ROS, the programmable release was triggered by an external low dose irradiation and the resulting enhanced local ROS lead to a remarkably increased therapeutic effect.

## 4 Conclusion and Outlook

This study demonstrated that hiROSponse^dox/ptx^ can elicit efficient tumor control effects as a programmable local drug delivery system in malignant melanoma models. The clinical approach presented here is the locoregional therapy of cutaneous/subcutaneous lesions or metastases of this tumor entity. In principle, the approach can also be applied to other tumor entities with nonsuperficial localization, then potentially assisted by ultrasound‐guided intratumoral administration. It is important to note that for tumor entities having a low local ROS level, the ROS‐responsive release can also be triggered by local external irradiation with a low dose. HiROSponse^dox/ptx^, featuring a programmable delivery mechanism for combination therapy, also fulfills many requirements for clinical application, including injectability, biocompatibility, biodegradability, as well as simple and large‐scale syntheses. HiROSponse can be further developed toward various inoperable solid tumor entities.

## 5 Experimental Section

### 5.1 Hydrogel Synthesis and Characterization

#### 5.1.1 Materials

For peptide synthesis, all required chemicals were purchased from IRIS Biotech GmbH (Marktredwitz, Germany) unless otherwise specified. Four‐arm polyethylene glycol (pentaerythritol) maleimide (maleimide functionalized starPEG) 10 kDa was bought from JenKem Technology (Beijing, China). Phosphate buffered saline (PBS) was bought from AppliChem GmbH (Darmstadt, Germany) as PBS powder. A Spectra/Por dialysis membrane, molecular weight cut off 8 kDa, was bought from Spectrum Laboratories, Inc. (Rancho Dominguez, CA, USA). TentaGel S RAM Fmoc (fluorenylmethyloxycarbonyl) rink amide resin was bought from Rapp Polymere GmbH (Tübingen, Germany). Peptide synthesis 6 mL columns and 5 mL syringes with included filters were bought from Intavis AG (Cologne, Germany). Polytetrafluoroethylene filter, polyvinylidene fluoride (PVDF) syringe filter, and filter holder were bought from Sartorius Stedtim (Aubagne, France). ProStar preparative high‐pressure liquid chromatography (HPLC) machine was bought from Agilent Technologies (Santa Clara, USA), and the AXIA 100A preparative C18 column (bead size 10 µm, 250 × 30 mm) from Phenomenex (Torrance, CA, USA). ResPep SL automated solid‐phase peptide synthesizer was purchased from Intavis (Cologne, Germany). ACQUITY analytical ultra HPLC (UPLC) with an ultraviolet light detector, the ACQUITY UPLC BEH analytical reverse phase C18 column (bead size 1.7 µm, 50 × 2.1 mm), and ACQUITY TQ electrospray ionization mass spectroscope (ESI‐MS) are from Waters (Milford MA, USA). The ALPHA 2–4 LD plus lyophilizer (Martin Christ Gefriertrocknungsanlagen GmbH, Osterode am Harz, Germany) was combined with the vacuum pump RZ6 (VACUUBRAND GmbH + Co KG, Wertheim, Germany). The MR Hei‐Standard stirring plate was purchased from Heidolph (Schwabach, Germany). 5(6)‐carboxytetramethylrhodamine (TAMRA) and phalloidin‐CF633 were bought from Thermo Fisher Scientific (Waltham, MA, USA).

#### 5.1.2 Peptide Synthesis

Peptides ((KA)_7_: CWGGKAKAKAKAKAKAKA) were prepared using standard fluorenylmethyloxycarbonyl (Fmoc) chemistry on a solid‐phase with 2‐(1H‐benzotriazole‐1‐yl)−1,1,3,3‐tetramethyluronium hexafluorophosphate (HBTU) activation on an automated solid‐phase peptide synthesizer. FITC was coupled via the same method with the N‐terminus of the peptides on the resin. The peptide was cleaved from the resin with TFA/TIS/water/DTT (90(v/v):5(v/v):2.5(v/v):2.5(m/v)) for 1.5 h. The product was precipitated and washed with ice‐cold diethyl ether.

#### 5.1.3 Peptide Purification

The peptide was dissolved in water containing 2 mg mL^−1^ TCEP. Peptide purification was performed via reverse‐phase HPLC on a preparative HPLC equipped with a preparative reverse‐phase C18 column. Purity was confirmed by analytical reverse‐phase UPLC using an analytical reverse phase C18 column, applying an isocratic gradient and electrospray ionization mass spectrometry.

#### 5.1.4 Synthesis of starPEG‐Peptide Conjugates

The synthesis of the peptide‐starPEG conjugates utilized in hydrogel assembly was conducted via Michael‐type addition reactions between maleimide‐terminated starPEG and cysteine‐terminated peptides from the library. Both components were dissolved in PBS (pH 7.4) with a total concentration of 80 mg mL^−1^. The reaction mixture was stirred on a stirring plate for 2 h. The crude product was analyzed by reverse‐phase UPLC using an analytical reverse‐phase C18 column and an isocratic gradient. The crude product was dialyzed to remove uncoupled peptides and salt in a dialysis tube with an 8 kDa cut‐off. Afterwards, the product was injected into the UPLC again to check its purity. The dialyzed product in water was lyophilized.

#### 5.1.5 Synthesis of Sulfated Hyaluronic Acid (S‐HA)

HA‐TBA was prepared as described previously.^[^ ^36^ ^]^ and then dissolved in DMF (5 mg mL^−1^) under argon air protection at room temperature. To produce the sulfated products, the first 1.5 g sulfur trioxide N,N‐dimethylformamide (SO_3_–DMF) complex was weighted. After dissolving it in 7 mL DMF, it was added into the HA‐TBA solution and stirred for 2 h. The resulting sulfated derivatives were dialyzed against a NaCl solution for 48 h, and then against deionized water for 5 days. The purified solution was frozen and lyophilized to obtain a dry product.

#### 5.1.6 Synthesis of Sulfated Hyaluronic Acid‐Ferrocene (S‐HA‐FeCp_2_)

HA‐TBA was prepared as described previously.^[^ ^36^ ^]^ and then dissolved in DMF (5 mg mL^−1^) under argon air protection at room temperature. To produce the S‐HA‐FeCp_2_, 100 mg of S‐HA‐TBA, 20 mg of ferrocenecarboxylic acid, and 10 mg of DMAP (dimethylaminopyridine) were added to the flask and purged with gaseous nitrogen before 25 mL of anhydrous DMSO were added to the vessel via cannulation. Then, the solution was stirred at 350 rpm for 1 h until the S‐HA‐TBA was fully dissolved. BOC_2_O (di‐tert‐butyl dicarbonate) was molten in a 37 °C‐warm water bath.

Then, 15.58 µL was added to the reaction using a plastic syringe. The reaction vessel was purged with nitrogen and stirred for 20 h at 45 °C. After the reaction mixture cooled down to RT, 10 mL of cold deionized water was added to quench the reaction and transfer the mixture to the dialysis membrane (6–8 kD). Dialysis was carried out for 5 days at room temperature while the water was changed twice per day. Then, the samples were frozen and lyophilized at −80 °C in a freezer for 12 h.

#### 5.1.7 Assembly of hiROSponse Hydrogel Without and With Drugs

S‐HA‐FeCp_2_ and peptide‐starPEG conjugates were dissolved at 2 × 10^−3^ m in MilliQ or full cell culture medium. Doxorubicin hydrochloride (dox, CAS 25316‐40‐9, LC Laboratories, USA) was dissolved in DMSO to give a 200 mg mL^−1^ stock. Paclitaxel (ptx, CAS 33069‐62‐4, LC Laboratories, USA was dissolved in DMSO to give a 150 mg mL^−1^ stock. S‐HA‐FeCp_2_ and peptide‐starPEG conjugates were dissolved at 2 × 10^−3^ m in MilliQ. Ptx was mixed with S‐HA‐FeCp_2_ and dox was mixed with peptide‐starPEG conjugates. Mixtures were vortexed for 30 s, and then slowly mixed for 2 h in tubes on the shaker. Both components (with or without drugs) were mixed in a 96 well plates (µ‐clear, Greiner Bio One, Frickenhausen, Germany) and formed hydrogels within 2 h for in vitro applications. Both components (with or without drugs) were mixed in 1 mL syringes or tubes and incubated for 6 h on the shaker before animal injection. Injection of 50 µL hydrogel in a mouse of 20 g bodyweight resulted in a dosage of 5 mg kg^−1^ dox and 5 mg kg^−1^ ptx.

#### 5.1.8 Drug Release In Vitro

The release of dox and ptx from hiROSponse^dox^, hiROSponse^ptx^ and hiROSponse^dox/ptx^ was investigated by dynamic dialysis. 500 µL of the hydrogel sample encapsulating the drugs were added to 1 mL PBS and placed in a dialysis bag with a molecular weight cutoff of 3.5 kDa. As control, dialysis of hiROSponse without drugs under the same conditions was performed. The dialysis bags were placed into centrifugal tubes containing 40 mL PBS and Tween‐80 (0.5%, w/w), with and without 0.2% hydrogen peroxide. They were incubated at 37 °C with mild waggle (100 rpm) subsequently. At predetermined time points (days 0, 1, 3, 5, 7, 10, 14), 200 µL of the supernatant from outside the dialysis bag was collected for analysis and centrifuged at 4 °C and 13000 rpm for 30 min. The supernatant was analyzed by HPLC.

#### 5.1.9 HPLC‐UV Method for Detection of Released dox and ptx

An LC system featuring an LC 10 ATVP pump and an Agilent injector (model 7125, 20 µL loop) was employed for the development and validation of chromatographic method. Chromatographic separation was achieved using an RP LiChrospher C18 column (100 × 4.6 mm, 5 µm; Merck) maintained at a column temperature of 35 °C. Both compounds were eluted under isocratic conditions with a mobile phase consisting of aqueous buffer and acetonitrile in a ratio of 37:63, respectively. The total flow rate of the mobile phase was 1 mL min^−1^, and the total runtime was 10 min. Detection of the eluent was performed at 231 nm using a Shimadzu SPD‐M10 UV‐PDA detector for both drugs.

#### 5.1.10 Contact Angle Tests

Contact angle tests were performed using an optical goniometer (DSA25S, Zeiss, Germany) at 25 °C to investigate the effect of ROS on the hydrophilicity of hiROSponse. The contact angles of 2 µL of water droplets on the sample surfaces were measured using a goniometer (DRI‐360, Shanghai, China).

### 5.2 Cell Culture

#### 5.2.1 Malignant Melanoma Cells

In this study, two different murine melanoma cell lines, more specifically malignant B16F10 cells and amelanotic B78H1 cells, were used for in vitro and in vivo experiments. B16F10 cells are highly metastatic tumor cells being derived from a C57BL/6J mouse. Over a total of 10 cycles, the F10 subclone of the B16 tumor line was generated by injecting B16 tumor cells into mice, collecting and culturing the resulting tumors, and re‐injecting them into new mice. The B78H1 cell line is a B16 subclone lacking tyrosinase activity, which was generated over 50 years ago.^[^ ^37^ ^]^ Amelanotic B78H1 cells were kindly provided by Prof. Pier‐Luigi Lollini and Dr. Lorena Landuzzi (University of Bologna). B16F10 and B78H1 cell samples were authenticated by ATCC using short tandem repeat (STR) analysis. Thereby, the submitted B16F10 sample profile was an exact match for the B16F10 (CRL‐6475) ATCC cell line in the ATCC mouse STR database. Regarding the STR analysis of the B78H1 sample, a common origin with B16F10 and other malignant melanoma cell lines (B16F0, B16F1) was confirmed with >75% match (Table S3, Supporting Information). Both cell lines were cultured in DMEM medium with 10 % fetal calf serum and 1 % penicillin/streptomycin (Biochrom AG) and incubated under standard cell culture conditions (37 °C, 5 % CO_2_, 95 % humidity).

#### 5.2.2 Spheroids

For the formation of spheroids, the hanging drop method was used by cultivating 3  ×  10^3^ B16F10 cells or 3  ×  10^4^ B78H1 cells in a total media volume of 25 µL containing 20 % methylcellulose for 4 days. Hereinafter, the spheroids were transferred to 96 well plates (total volume of 200 µL DMEM per well) comprising either 5 µL hiROSponse or hiROSponse^dox/ptx^, 0.1 % H_2_0_2_ (positive control for ROS generation), 1 × 10^−6^ m RSL‐3 (ferroptosis inducer), 10 × 10^−6^ m Liproxstatin‐1 (ferroptosis inhibitor), 50 × 10^−6^ m Z‐VAD‐FMK (apoptosis inhibitor), or 0.5 mg mL^−1^ dox/ptx. Next day, spheroids were irradiated with 2 Gy prior to picking them for further analysis by flow cytometric (vital 3D cultures) or qPCR (snap‐frozen samples) analysis.

#### 5.2.3 Determination of Melanin Concentration

To verify the difference in melanin content between B16F10 and B78H1 cells, pellets of both melanoma cell lines were gained by detaching the cells from cell culture flasks using trypsin‐EDTA solution. Subsequently, the obtained cell solutions were counted using CASY cell counter (Omni Life Science GmbH & Co. KG). After centrifugation (300 ×  g, 3 min), the cell pellets were dissolved in 1 N NaOH containing 10 % DMSO and incubated by shaking at 80 °C for 2 h. All samples were centrifuged at 12 000 ×  *g* for 10 min at room temperature and the resulting supernatants were used for photometric analysis.^[^ ^38^ ^]^ In comparison to a standard curve created by synthetic melanin (Fisher Scientific), the melanin concentration was determined by absorbance measurement at 470 nm using Cytation 5 (BioTek).

### 5.3 Animal Experiments

#### 5.3.1 Hydrogel Injection

Animal experiments were conducted according to the guidelines of German Regulations for Animal Welfare. The underlying protocols were approved by the local Ethical Committee for Animal Experiments (reference numbers DD24.1‐5131/450/16, DD24.1‐5131/449/49, 25–5131/496/34). For initial degradation and biocompatibility investigations, female immunocompetent hairless Crl:SKH1‐Hrhr mice were purchased from Charles River and housed at 27 ± 1 °C and 55 ± 10 % humidity with a 12 h light cycle. Pre‐gelated hydrogels (50 µL) were subcutaneously injected by a 1 mL syringe and a 27G needle in the lower back area of each SKH1 mouse (age 7–8 weeks, weight 20–27 g) being anesthetized with 10 % (v/v) desflurane (Baxter).^[^ ^12^, ^18^ ^]^ Regarding tumor control experiments, female immunocompetent C57BL/6JRj mice were purchased from Janvier Labs and housed at 22 ± 2 °C and 55 ± 10 % humidity with a 12 h light cycle. To generate a syngeneic tumor‐bearing mouse model, 1 × 10^5^ B16F10 cells (passage 10–15) or 1 × 10^6^ B78H1 cells (passage 31–36) were injected subcutaneously in the right lower leg of each C57BL/6JRj mouse (age 8–15 weeks, weight 17–24 g) being anesthetized with 10 % (v/v) desflurane. After 7 days, when tumors reached a size of about 20 mm^3^ (B16F10, range: 1–100 mm^3^) and 10 mm^3^ (B78H1, range: 3–30 mm^3^), animals were randomized to the groups and 50 µL of the pre‐gelated hydrogels were injected into or as closely as possible to the initially grown tumor by a 1 mL syringe and a 27G needle. Additionally, the local tumor area was irradiated with 2 Gy using a Maxishot X‐ray system having a Y.TU/320‐D03 tube (YXLON) at d0 and d3 after hydrogel injection to generate ROS in the TME by external irradiation.

#### 5.3.2 In Vivo Hydrogel and Tumor Volume Measurements

Hydrogel and tumor volume as well as inguinal lymph node size were measured using dedicated 7T small animal MRI device (ParaVision software 6.0.1., Bruker). For MRI measurements, a T2‐weighted measuring sequence (TRARE) was applied with an echo and repetition time of 38 and 4,300 ms, respectively. Spatial resolution was set to 150 µm in *xy*‐direction. The slice thickness was set to 0.8 mm. Hereinafter, hydrogel and tumor volumes as well as lymph node sizes were quantified using the software ROVER (version v3.0.57 h; ABX GmbH). As already described,^[^ ^12c^ ^]^ TPA (12‐O‐tetradecanoylphorbol‐13 acetate) injected and untreated animals served as positive and negative controls for comparison of the inguinal lymph node sizes, respectively. Moreover, survival plots (Kaplan–Meier curves) were generated by GraphPad Prism software (version 7). Therefore, an exponential growth was modeled based on the tumor volume measurements and the calculated 100‐fold increase of the respective initial tumor volume was defined as termination criterion after extrapolation (Figures S4A and S14, Supporting Information).

### 5.4 Ex Vivo Detection of ROS Using a Fluorogenic Reagent

Since the quantification of ROS analysis in vivo was limited, the ROS level was additionally investigated ex vivo by flow cytometry. Tumor samples as well as spheroids were investigated in terms of the presence of ROS using CellROX Deep Red Reagent (Invitrogen). Therefore, B16F10 tumor samples were resected 3 days after the start of the experiments (d0 reflecting the day of hydrogel injection) and spheroids were analyzed 1 day after starting the experiment (d0 reflecting the day of transferring the spheroids to hydrogel‐containing 96 well plates). Extracted B16F10 tumor samples were separated through a 40 µm cell strainer (Bel‐Art), whereas B78H10 tumor samples had to be crushed manually by scissors and pushed through a cell strainer with a stamp as the amelanotic tumors were tighter and more compact compared to the melanotic specimens. Tumor cell solutions and spheroids were incubated with 5 × 10^−6^ m CellROX at 37 °C for 90 min, hereinafter. Additionally, tumor samples were stained with a Viobility 405/520 Fixable Dye (Miltenyi Biotec, 1:100) at 37 °C for 15 min to discriminate between live and dead cells as the separation process may increase the percentage of dead cells to be gated out in flow cytometric analysis. Prior to flow cytometric analysis of the fluorogenic probe, single cell solutions of the melanoma spheroids were obtained by incubation with 2 × 10^−3^ m EDTA for 5 min at 37 °C. All samples were resuspended in measuring buffer containing 2% FCS, 5 × 10^−3^ m EDTA and 0.01% sodium azide in PBS. Afterwards, fluorescence (excitation 638 nm, emission 650–670 nm) was measured by Attune NxT Acoustic Focusing Cytometer and the percentage of positively stained cells was determined according to gating strategy shown in Figure S13A,B (Supporting Information).

### 5.5 Investigation of Drug Release Kinetics Using Murine Blood Plasma Samples

For the investigation of the drug release kinetics, blood samples were taken from tumor‐bearing mice by venous blood sampling from the tail vein (d1, d3, and d7) or final cardiac puncture at the endpoint of the experiment with heparin‐wetted cannulas. An aliquot of these blood samples was used to count the WBCs by CASY cell counter as a low WBC count is a certain side effect during cancer treatment. The remaining blood samples were centrifuged at 2000  ×  *g* at 4 °C for 15 min to obtain blood plasma for the drug release study being stored at −70 °C until further analysis. To measure the concentration of dox and ptx by mass spectrometry, blood plasma samples were pooled (three mice per group), mixed with acetonitrile (sample/acetonitrile 1/7 v/v) and incubated on ice for 10 min to precipitate the proteins. After centrifugation for 1 min at 2000 g, the supernatants were withdrawn and directly analyzed by reverse‐phase UPLC‐MS/MS in multiple reaction monitoring (MRM) mode. Samples were analyzed in duplicates by injecting 2 or 5 µL. The drug concentration was determined according to drug standard/calibration curves (Figures S18 and S19, Supporting Information).

Analyses were performed with ACQUITY UPLC I class including an ACQUITY UPLC PDA eλ detector coupled to a Xevo TQ‐S mass spectrometer. An ACQUITY Premier, Peptide BEH C18 column (1.7 µm, 300 Å, 100 × 2.1 mm, equipped with an ACQUITY Premier, Peptide BEH C18 VanGuard Pre‐column, 1.7 µm, 300 Å, 5 × 2.1 mm) was used as stationary phase. A binary gradient system of 0.1% CH_3_COOH/water (solvent A) and 0.1% CH_3_COOH in CH_3_CN/CH_3_OH (1:1, v/v, solvent B) at a flow rate of 0.4 mL min^−1^ served as the eluent as follows: for ptx (t_0 min_ 55/45 – t_0.5 min_ 55/45 – t_5.5 min_ 5/95 – t_7 min_ 5/95 – t_8.0 min_ 55/45 – t_8.5 min_ 55/45) and dox (t_0 min_ 75/25 – t_0.5 min_ 75/25 – t_5.5 min_ 25/75 – t_6.0 min_ 5/95– t_7 min_ 5/95 – t_8.0 min_ 75/25 – t_8.5 min_ 75/25). Ionization was carried out in the Zspray Ionization chamber using electrospray ionization in positive mode. The set up for the Xevo TQ‐S MS detector was 150 °C source temperature, 500 °C desolvation temperature, 150 L/h cone gas, and 1000 L/h desolvation gas (nitrogen). Optimized transitions for MRM were found to be as follows: dox (544.17→397.09 at Collision energy (CE) 10, Cone Voltage (CV) 6; 544.17→361.07 CE 10, CV 2) and ptx (854.27→104.98 CE 64; CV 30; 854.27→286.10 CE 22, CV 30). The evaluation of the UPLC chromatograms and mass spectra was performed with massLynx (v4.1) and TargetLynx (v4.1) software.

For quantification, the calibrated range was chosen in the linear range and so that deviations at LLOQ were smaller than 10%, being 0.5‐100 fmol on column (fmol o.c.) for dox and ptx, respectively. With the chosen sample workup and injection volume, this resembles a LLOQ of dox and ptx in plasma of 2 and 0.8 × 10 ^−9^ m, respectively. Transitions used for quantification were 544.17→361.07 (dox) and 854.27→286.10 (ptx), with 544.17→397.09 (dox) and 854.27→104.98 (ptx) as qualifiers. The mean extraction recovery rates of ptc and dox from spiked blood at 5 × 10 ^−9^ m were greater 95%. The recovery from blood stored at −80 °C was consistent over the maximum storage time of 3 months.

### 5.6 Analysis of Murine Urine Samples Regarding Protein Concentration and pH Value

An increased albumin excretion via the urinary tract may indicate impaired renal function. In order to rule out this side effect, spontaneous urine of the tumor‐bearing mice was collected and further analyzed photometrically. Therefore, three mice per group were placed in a cage without bedding, the urine was collected at certain days (d1, d7, d10, or d14 after hydrogel injection) and stored at −70 °C until further analysis. The pH values of urine samples were determined using pH indicator strips (Figure S11, Supporting Information, Carl Roth). Additionally, urine samples were investigated regarding the protein concentration measured by bicinchoninic acid protein assay (Thermo Fisher Scientific). Thereby, bovine serum albumin served as standard reference while measuring the absorption at 562 nm after an incubation at 37 °C for 30 min.

### 5.7 Ex Vivo Histological Stainings

#### 5.7.1 Sample Extraction and Histological Overview Staining

Histological and immunohistochemical analysis of surgically extracted tissue samples were accomplished as described previously.^[^ ^39^ ^]^ to study local tissue responses at the hydrogel‐tissue interface defining the hydrogel biocompatibility in the SKH1 mouse model and to investigate molecular characteristics of B16F10 tumors of the melanoma‐bearing C57/BL6 mouse model. In brief, the mice were sacrificed by cervical dislocation and the remaining hydrogels with the surrounding tissue (at day 25 after hydrogel injection) as well as certain organs (liver, kidney, spleen, lymph nodes) and tumor samples were removed. Afterwards, extracted tissue specimen were fixed in 4% (v/v) PFA for 24 h. Subsequently, organ and skin samples were incubated in 0.1% sodium azide and 20% (w/v) sucrose in PBS for 3 days at room temperature, respectively. For cryosections, skin samples were bisected in the middle of the remaining hydrogel and embedded in 7.5% (w/v) gelatin and 20% (w/v) sucrose in PBS. After freezing, the gelatin‐embedded samples were cut to 5 µm sections in a cryostat at −30 °C (Leica CM1850). On the other hand, tumor and organ samples were dehydrated in an increasing isopropanol row and RotiClear, followed by embedment in paraffin. Paraffin‐embedded tissue samples were sectioned (slice thickness of 2 µm) using a rotary microtome (MICROM International GmbH). Prior to immunohistochemical staining, tissue sections had to be deparaffinized to make the antigens accessible. Therefore, the tissue sections were heated at 60 °C for 30 min in a dry cabinet, incubated in RotiClear (Carl Roth GmbH & Co. KG) and in a descending ethanol series afterwards. For histological analysis, a hematoxylin & eosin (H&E) stain and a Van Gieson's stain to study the constitution and thickness of subcutaneous connective tissue layers indicating fibrotic tissue alterations were conducted as described previously using standard protocols.^[^ ^12^ ^]^ In brief, tissue sections were stained for 3 min in hematoxylin staining solution according to Mayer. After blueing, sections were stained in eosin B solution for 30 s and, hereinafter, eosin was fixed in an increasing ethanol series. Sections were incubated for 5 min in RotiClear and covered with RotiMount. In terms of Van Gieson's stain, elastin fibers were stained by incubation in resorcin‐fuchsin solution (Carl Roth GmbH & Co. KG) at 60 °C for 30 min. This was followed by nuclear staining with Weigert's ferric hematoxylin solution for 5 min at room temperature. The section sections were then incubated in Van Gieson mixture consisting of picric acid (AppliChem GmbH) and 1% (v/v) thiazine red (MORPHISTO GmbH) to stain collagen fibers. Finally, the sections were covered with RotiMount. Regarding the measurement of the thickness of subcutaneous connective tissue layers, for each section five points on each site of the remaining hydrogel, directed either to the skin or muscular site, were recorded by AxioImager.A1 microscope and measured with the corresponding AxioVision software (Carl Zeiss, version 4.8).

#### 5.7.2 Immunohistochemical Staining of Tissue Marker Proteins

Additionally, specific tissue response was visualized using immunohistochemical staining for several inflammation marker, pan‐macrophages, matrix remodeling, angiogenesis, proliferating cells as well as epithelial and mesenchymal marker. Briefly, an antigen retrieval in 10 × 10^−3^ m heated citrate buffer in four heating cycles (except for CD68) was performed, followed by quenching of endogenous peroxidase and endogenous biotin in 3% (v/v) hydrogen peroxide for 10 min and by using Biotin‐Blocking System from Dako according to manufacturer's instructions, respectively. Tissue sections were incubated in 10% (v/v) FCS or 5% BSA and 1% Tween (only for CD44, EpCAM) in TBS‐T for 1 h to block unspecific binding and, hereinafter, incubated with primary antibody or isotype control (**Table** **1**) at 4 °C overnight. An incubation with a biotinylated secondary antibody against rabbit or rat (Table 1) for 1 h followed. For visualization, sections were incubated with ExtrAvidin peroxidase (Sigma Aldrich, 1:50) for 30 min and AEC substrate kit (BD Biosciences, 1:50) for 2–5 min. Tissue sections were counterstained with Mayer's hematoxylin, embedded in aqueous solution and imaged using AxioImager.A1 microscope with AxioVision software (version 4.8, Carl Zeiss). Immunohistochemical staining was quantified using ImageJ/FIJI (version 1.52i; National Institutes of Health). Therefore, a color threshold plugin was used. RGB values were set for cell nuclei and immunohistochemical positively stained areas. After applying the analyze particles plugin, positively stained area was divided by cell nuclei area.

**Table 1:** Antibodies for immunohistochemical staining.

Antibody	Company	Catalogue number	Species	Dilution	RRID
Primary antibody					
Inflammation marker					
COX‐2	Abcam	ab15191	Rabbit	1:500	AB_2085144
RAGE	Santa Cruz Biotechnology	sc‐5563 (H300)	Rabbit	1:200	AB_2224490
TM	Santa Cruz Biotechnology	sc9162	Rabbit	1:50	AB_2201929
Pan‐macrophages					
CD68	AbD Serotec	MCA‐1957	Rat	1:100	AB_322219
Matrix remodeling					
TG‐2	Santa Cruz Biotechnology	sc‐20621	Rabbit	1:50	AB_793379
Angiogenesis					
VEGF	Santa Cruz Biotechnology	sc152	Rabbit	1:100	AB_2212984
CD31	Abcam	ab28364	Rabbit	1:75	AB_726362
Proliferation					
Ki67	Abcam	ab15580	Rabbit	1:200	AB_443209
Epithelial marker					
KRT19	Abcam	ab52625	Rabbit	1:600	AB_2281020
EpCAM	Abcam	ab124825	Rabbit	1:100	AB_10973714
Laminin	Abcam	ab11575	Rabbit	1:500	AB_298179
Occludin	Thermo Fisher Scientific	71‐1500	Rabbit	1:100	AB_2533977
Mesenchymal marker					
CD44	Abcam	ab157107	rabbit	1:2000	AB_2847859
Fibronectin	Abcam	ab2413	rabbit	1:50	AB_2262874
α‐SMA	Abcam	ab5694	rabbit	1:300	AB_2223021
Isotope control					
Normal rat IgG	Santa Cruz Biotechnology	sc‐2026	rat	Concentrations comparable to primary antibody	AB_737202
Rabbit polyclonal IgG	Abcam	ab27478	rabbit	Concentrations comparable to primary antibody	AB_2616600
Secondary antibody (biotinylated)					
Anti‐rabbit IgG	Dianova	111‐065‐003	goat	1:200	AB_2337959
Anti‐rat IgG	Dianova	312‐066‐045	rabbit	1:200	AB_2339843

### 5.8 Investigation of Ferroptosis

#### 5.8.1 TBARS Assay

To study lipid peroxidation, a TBARS (thiobarbituric acid reactive substances) assay (Cell Biolabs) was used to detect oxidative damage in tumor samples extracted 3 days after hydrogel injection in comparison to a MDA standard. TBARS assay was performed according to manufactures’ instructions. In brief, all samples were mixed with butylated hydroxytoluene in order to prevent additional oxidation. For tissue homogenization, metal beads were deployed and lysis was done for 3 × 3 min at 25 Hz. All samples were spinned at 15 000 × g for 15 min to collect the supernatant. Afterwards, 100 µL SDS lysis solution was added and the samples were incubated for 5 min at room temperature. After adding 250 µL TBA reagent, another incubation step at 95 °C for 60 min followed. Once the samples cooled to room temperature (4 °C for 5 min), the samples were centrifuged at 3000 rpm (0.9 × g) for 15 min. For preventing the interference of hemoglobin, a butanol extraction was done. Therefore, the supernatants (300 µL) were mixed with 300 µL *n*‐butanol, vigorously vortexed for 1–2 min and centrifuged at 10 000 × *g* for 5 min. Finally, the samples (150 µL) were measured fluorometrically in duplicates with an excitation and emission of 540 nm and 590 nm, respectively.

#### 5.8.2 8‐Hydroxy‐2‐Deoxyguanosine Assay

For tissue homogenization, Wizard Genomic DNA Purification Kit (Promega) was used according to manufacturer's instructions. Briefly, 25 mg tumor tissue was mixed with 750 µL Nuclei Lysis Solution in M tubes (Miltenyi). By applying program RNA_02_01, tissue homogenization was done for 2 min using GentleMACS (Miltenyi). Afterwards, M tubes were centrifuged at 2000 × g for 1 min and lysates were incubated at 65 °C for 15–30 min. Further, 3 µL RNase Solution was added to the lysates, mixed, and incubated at 37 °C for additional 15–30 min. After the samples reached RT, 200 µL protein precipitation solution was added and the samples were vortexed for 20 s. An incubation on ice for 5 min followed. Hereinafter, samples were centrifuged at 15000 × *g* for 4 min and the supernatants were mixed with 600 µL isopropanol. An additional centrifugation step at 15000 × *g* for 1 min followed. An amount of 600 µL of 70% ethanol was added to the white pellets to wash the DNA. Samples were centrifuged again as described earlier. Lastly, 100 µL DNA rehydration solution was added to the dried pellets and the samples were incubated at 65 °C for 1 h. DNA samples were stored at 2–8 °C and further used for 8‐OHdG assay. Prior to the assay, samples were incubated with Nucleoside Digestion Mix (New England Biolabs) at 37 °C for 1 h, boiled for 10 min, and placed on ice. 8‐OHdG assay (ab201734, Abcam) was conducted according to manufacturer's instructions. Prepared samples and 8‐hydroxy‐2‐deoxyguanosine standard were mixed with the prepared 8‐OHdG antibody. Hereinafter, the plate was covered and incubated for 1 h at room temperature. Four washing cycles are following. Furthermore, 100 µL TMB substrate were added to the samples and the plate was incubated at room temperature for 30 min in the dark to allow the enzymatic color reaction to develop. Finally, the reaction was stopped by adding 100 µL stop solution and the absorbance was measured at 450 nm using the microplate reader Cytation 5 (BioTek).

#### 5.8.3 qPCR

Prior to qPCR experiments, RNA was extracted from spheroid samples using miRNeasy Tissue/Cells Advanced Mini Kit (Qiagen) according to manufactures’ instructions. In brief, RLT buffer with β‐mercaptoethanol was added to spheroid pellets. Homogenization was achieved by vortexing samples and applying 15 s ultrasound. Afterwards, 140 µL AL buffer were added and samples were incubated at room temperature for 3 min. Homogenized lysates were transferred to gDNA Eliminator spin columns and centrifuged at 8000 ×  g for 30 s. Isopropanol (590 µL) was added to the flow‐through. Hereinafter, samples were transferred to RNeasy Mini columns and spinned at 8000 ×  g for 15 s. Further, 350 µL RWT buffer was added and another centrifugation step as described earlier followed. DNase incubation mix (80 µL), containing DNase (RNase‐free DNase set, QIAGEN) diluted with RDD buffer, was added to the samples and the loaded columns were incubated at room temperature for 15 min. Additional wash steps using RWT buffer, RPE buffer, and 80 % ethanol as well as intermittent centrifugation steps followed as described above. RNA samples were eluted by adding 40 µL RNase‐free water and centrifugation (1 min, full speed). RNA content was determined using NanoPhotometer NP80 touch (IMPLEN GmbH). For qPCR experiments, qPCR kit SYBRgreen (QIAGEN) and primer (EUROTINS GENOMICS, **Table** **2**, for annealing and melting curves see Figure S20, Supporting Information) were used.

**Table 2:** Primer used for qPCR.

Gene	Forward primer (5′ → 3′)	Reverse primer (5′ → 3′)
Ferroptosis		
GPX4	TAAGAACGGCTGCGTGGTGAAG	AGAGATAGCACGGCAGGTCCTT
ACSL4	CCTGAGGGGCTTGAAATTCAC	GTTGGTCTACTTGGAGGAACG
TFR1	GCTCCGAGGACTTTCGTCGT	AGGGCTGATTCCAAGGGTGT
SLC7A11	TCGAGTCTGGGTGGAACTGC	ACTCCAGCTGACACTCGTGC
Apoptosis		
APAF1	GCTCTGCCTTCTCGCTGGAT	CCGAGATCGGAGCACACGAA
CASP9	ACATCGAGACCTTGGATGGCA	ACAGCATTGGCAACCCTGAG
CASP3	GGAGCTTGGAACGGTACGCT	AGTCCACTGACTTGCTCCCA

During qPCR, the expression of housekeeping genes was monitored using the Mouse Housekeeping Genes Primer Set (Biomol) RNA samples and primer were diluted with RNase free water to a final concentration of 50 ng RNA and 2 × 10^−6^ m primer, respectively. Afterwards, reaction mix containing QuantiTec SYBR Green RT‐PCR Master Mix, forward primer, reverse primer, QuantiTec RT Mix, RNase‐free water, and template RNA was prepared and mixed thoroughly. qPCR was performed according to cycling conditions mentioned in **Table** **3** using Bio‐Rad CFX device with Bio‐Rad CFX manager software.

**Table 3:** qPCR cycling conditions of the 40 cycles performed.

Step	Time	Temperature [°C]
Reverse transcription	30 min	50
PCR initial heat activation	15 min	95
3‐step cycling		
Denaturation	15 s	94
Annealing	30 s	58
Extension	30 s	72
Data acquisition	15 s	77.5

### 5.9 Statistical Analysis

Pre‐processing qPCR data, the control group (medium) served as reference and was set to 2^−ΔΔCt^ = 1. Further, quantified hydrogel and melanoma tumor volumes were normalized to day 0 as indicated in the respective figure legends. Using GraphPad Prism software (version 9.3.1.) for further analysis, descriptive parameters of the different tumor growth profiles, such as doubling time and tumor growth rate, and hydrogel degradation were calculated by exponential growth model and linear regression model, respectively. In addition, correlation analysis between tumor and hydrogel volumes was done computing Pearson correlation coefficients (*r*) with two‐tailed *p* values and 95% confidence interval. In order to determine the drug concentrations of dox and ptx in murine plasma samples, a linear regression of drug standard/calibration curves was performed. For analysis of statistical significance, a one‐way ANOVA followed by a Bonferroni post‐hoc test, two‐tailed unpaired *t*‐test, or, in terms of survival analysis, a log‐rank test was applied. *p* values ≤ 0.05 were considered as statistically significant. Figure legends contain information on sample size, presented data mean with errors (SEM/SD), *p* values, and the specific statistical test.

## Conflict of Interest

The authors declare no conflict of interest.

## Supporting information

Supporting Information

Supporting Information

## Data Availability

The data that support the findings of this study are available from the corresponding author upon reasonable request.
