# Supplementary material for: Programmable Release of Chemotherapeutics from Ferrocene‐Based Injectable Hydrogels Slows Melanoma Growth
Source: Adv Healthc Mater. 2024 Jul 15;13(27):2400265. doi: 10.1002/adhm.202400265 (PMC12344625; doi:10.1002/adhm.202400265)
Supplement: Supplementary file 1 — Supporting Information [file ADHM-13-0-s001.pdf]

ADVANCED HEALTHCARE MATERIALS

Supporting Information

for *Adv. Healthcare Mater.*, DOI 10.1002/adhm.202400265

Programmable Release of Chemotherapeutics from Ferrocene-Based Injectable Hydrogels Slows Melanoma Growth

Rebecca Rothe, Yong Xu, Johanna Wodtke, Florian Brandt, Sebastian Meister, Markus Laube, Pier-Luigi Lollini, Yixin Zhang, Jens Pietzsch* and Sandra Hauser**

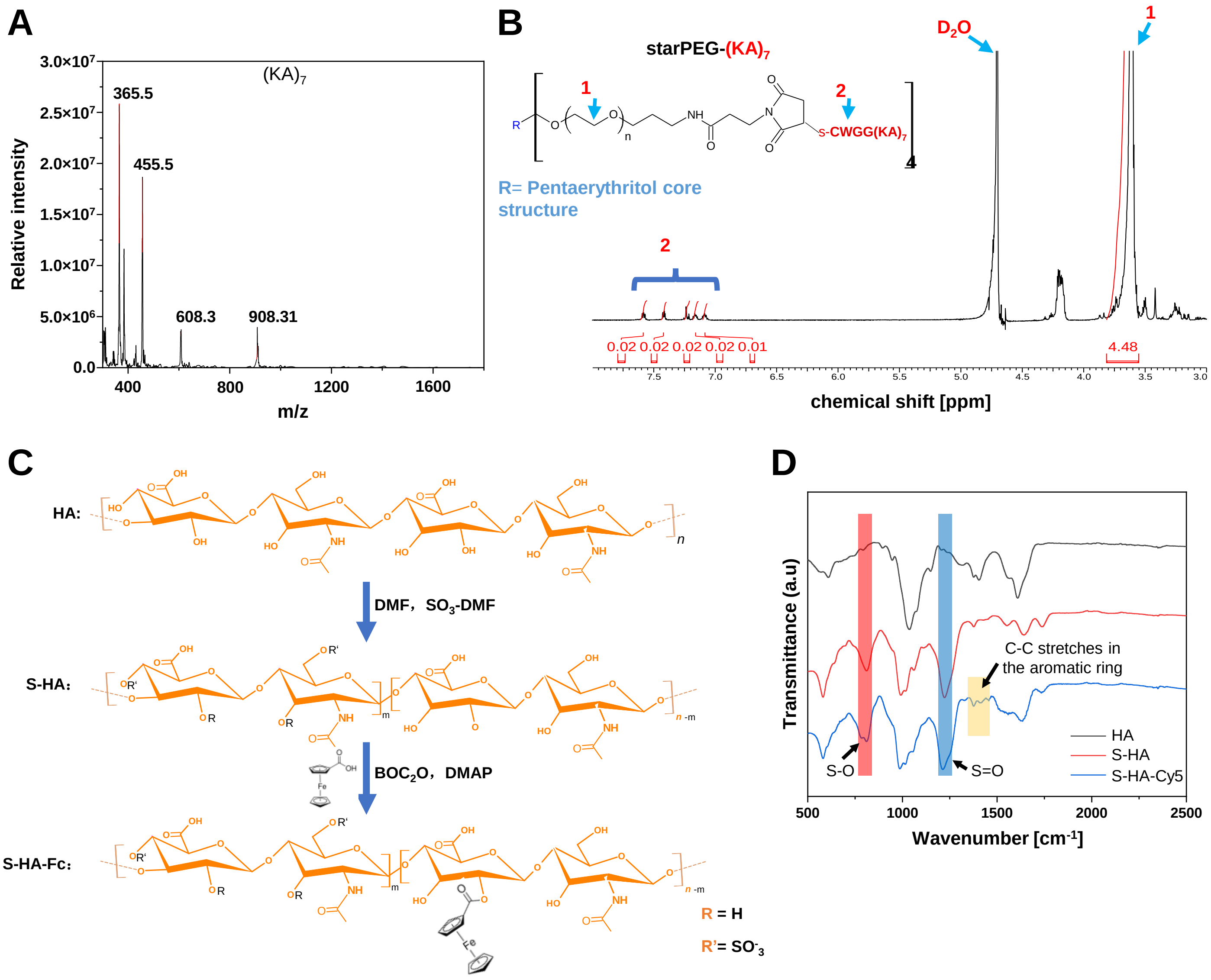

Figure S1A. Mass spectrometer (MS-ESI, positive mode) graphs of synthesized peptides. **B.** Characterization of starPEG-$(KA)_7$ by ^{1}H-NMR. $(KA)_7$ and starPEG conjugation was confirmed via tryptophan (W) aromatic peaks (δ = 7.09, 1H; δ = 7.16, 1H; δ = 7.42, 1H ; δ = 7.58, 1H;) and PEG ethyl peak (δ = 3.62, 2H). **C.** Scheme of the synthesis of S-HA and S-HA-$FeCP_2$ polymers. **D.** ATR-FTIR spectra of HA, S-HA, and S-HA-$FeCP_2$.

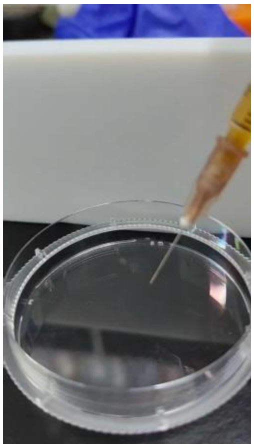

Movie S1. Injection of hiROSponse via 27G needle.

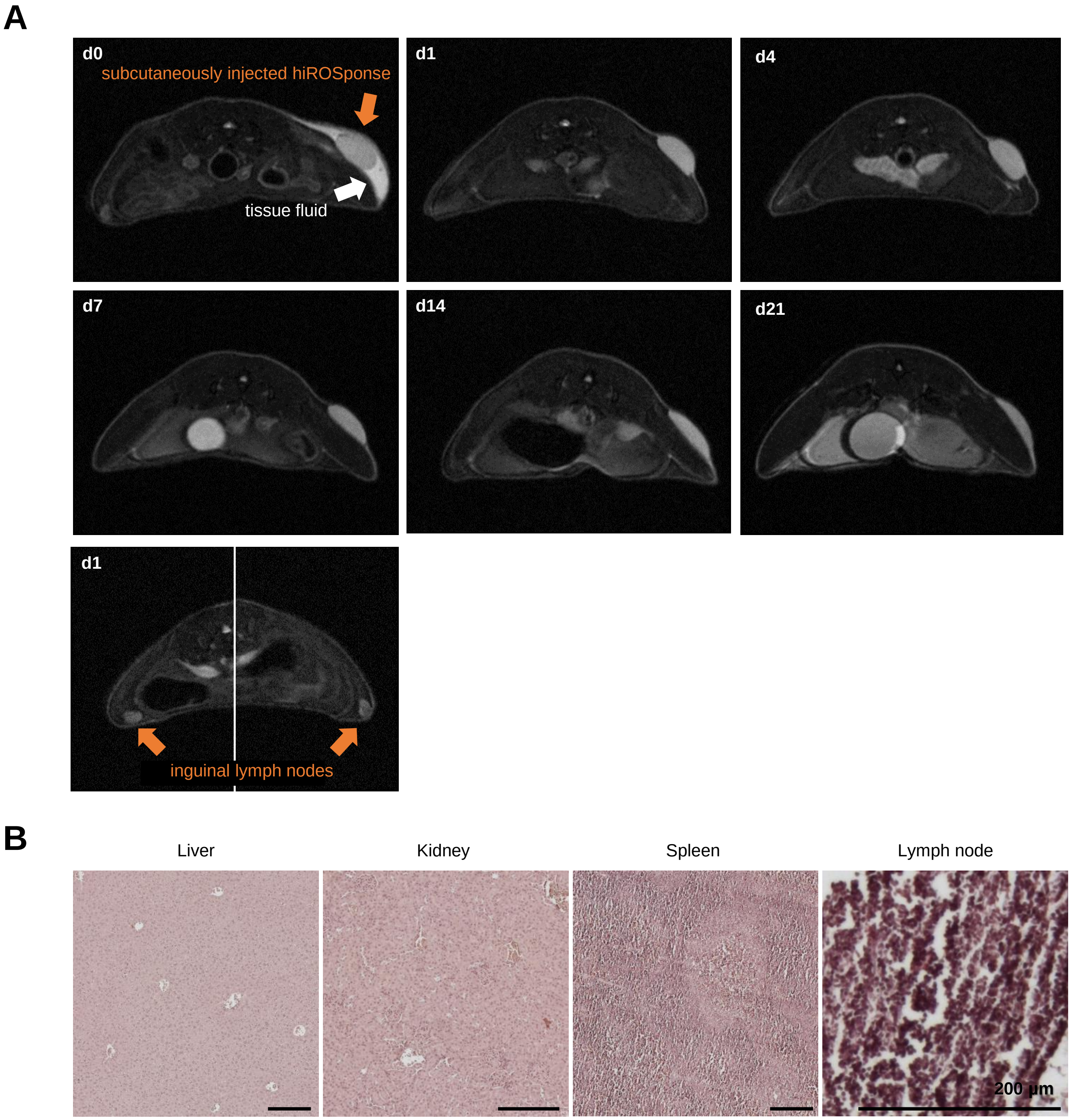

Figure S2: **Characterization of hiROSponse in healthy SKH1 mice. A.** Axial MRI images of subcutaneously injected hiROSponse (day 0 with accumulated tissue fluid surrounding the hydrogel, day 1 without swelling, ongoing degradation until day 21) and inguinal lymph nodes. **B.** Representative H&E stainings of paraffin-embedded organs, which have been dissected from animals with subcutaneously injected hydrogel at day 25.

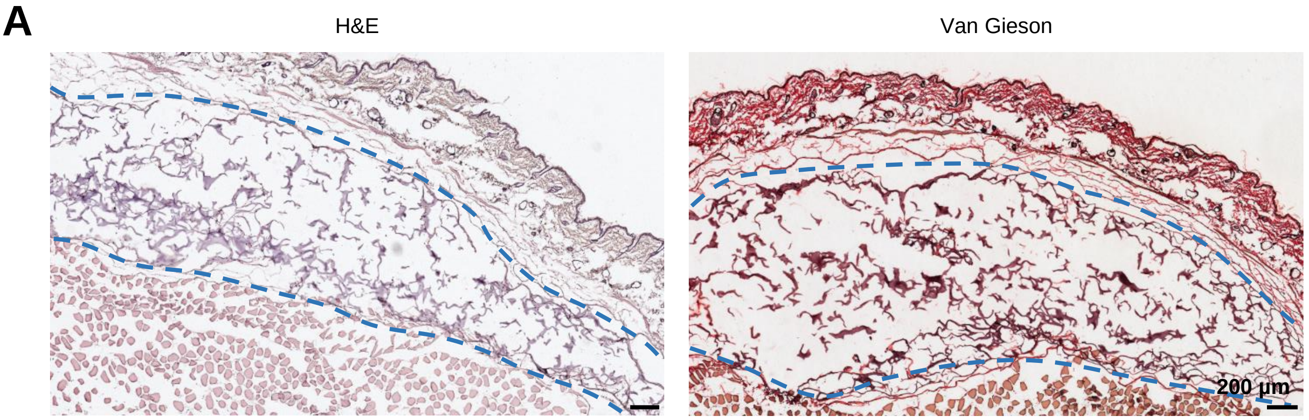

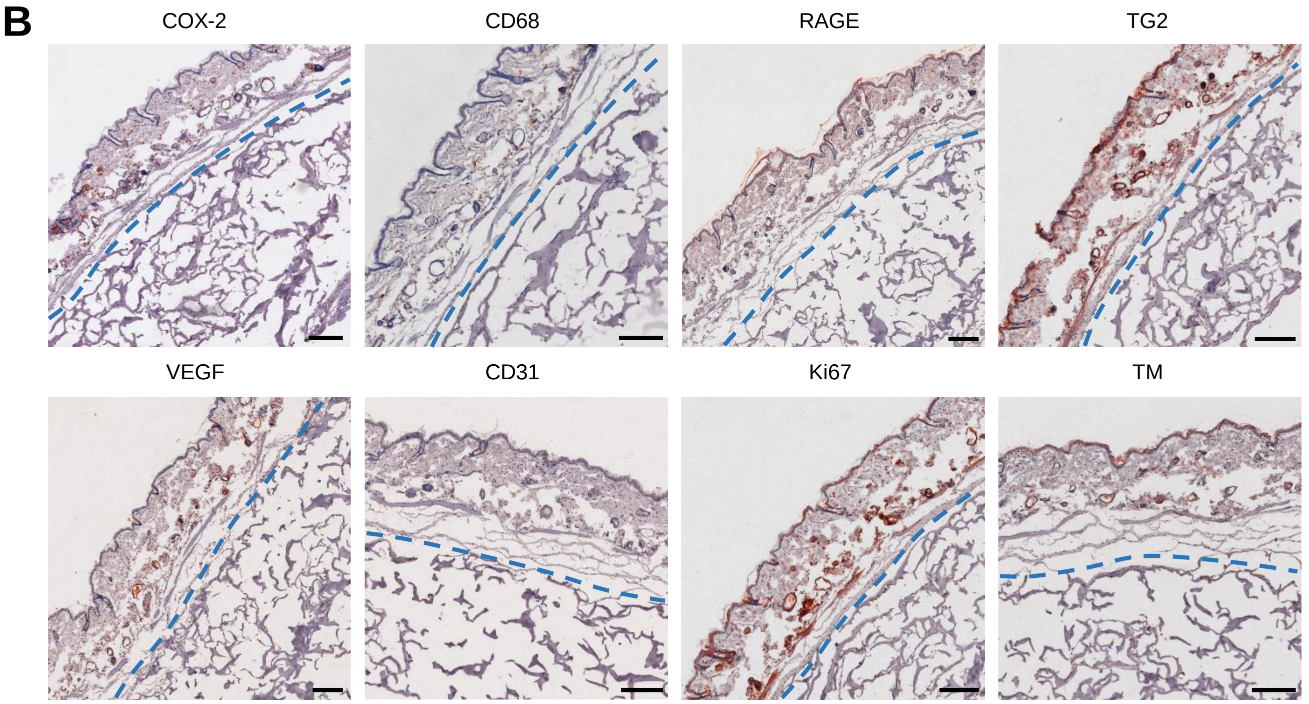

Table S1: Quantification of immunohistochemical stainings and thickness of the subcutaneous connective tissue layers (mean ± SD)

	hiROSponse	negative control
COX-2	0.11 ± 0.09	0.80 ± 0.22
CD68	0.09 ± 0.05	0.02 ± 0.02
RAGE	0.28 ± 0.15	0.11 ± 0.04
TG-2	0.27 ± 0.04	0.18 ± 0.11
VEGF	0.15 ± 0.04	1.77 ± 0.85
CD31	0.01 ± 0.01	0.07 ± 0.04
Ki67	0.88 ± 0.32	0.33 ± 0.19
TM	0.09 ± 0.04	0.09 ± 0.06
thickness of subcutaneous connective tissue	247.39 ± 65.52 µm	244.89 ± 46.00 µm

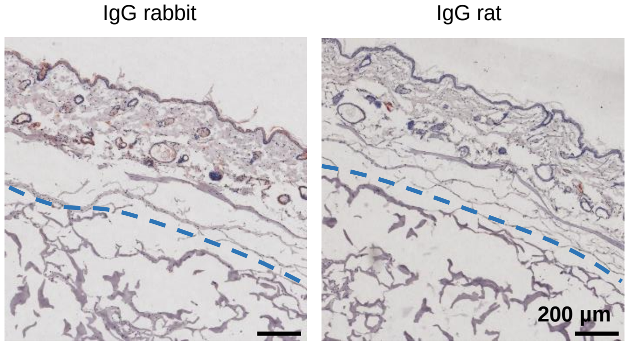

Figure S3: **Hydrogel-tissue interface of hiROSponse in healthy SKH1 mice**. **A.** Representative histological H&E staining (left) and Van Gieson's staining (right) for visualization of elastin and keratin fibers to determine the thickness of the subcutaneous connective tissue. **B.** Representative immunohistochemical stainings of markers for inflammation (COX-2, TM, RAGE), pan-macrophages (CD68), matrix remodeling (TG-2), angiogenesis (VEGF, CD31), and proliferation marker Ki67, cell nuclei in blue and positive immunohistological staining in red, blue line indicates hydrogel-tissue interface. **Table S1:** Quantification of immunohistochemical stainings (positive stained area related to cell nuclei area) and thickness of the subcutaneous connective tissue layers, n = 3, mean ± SD.

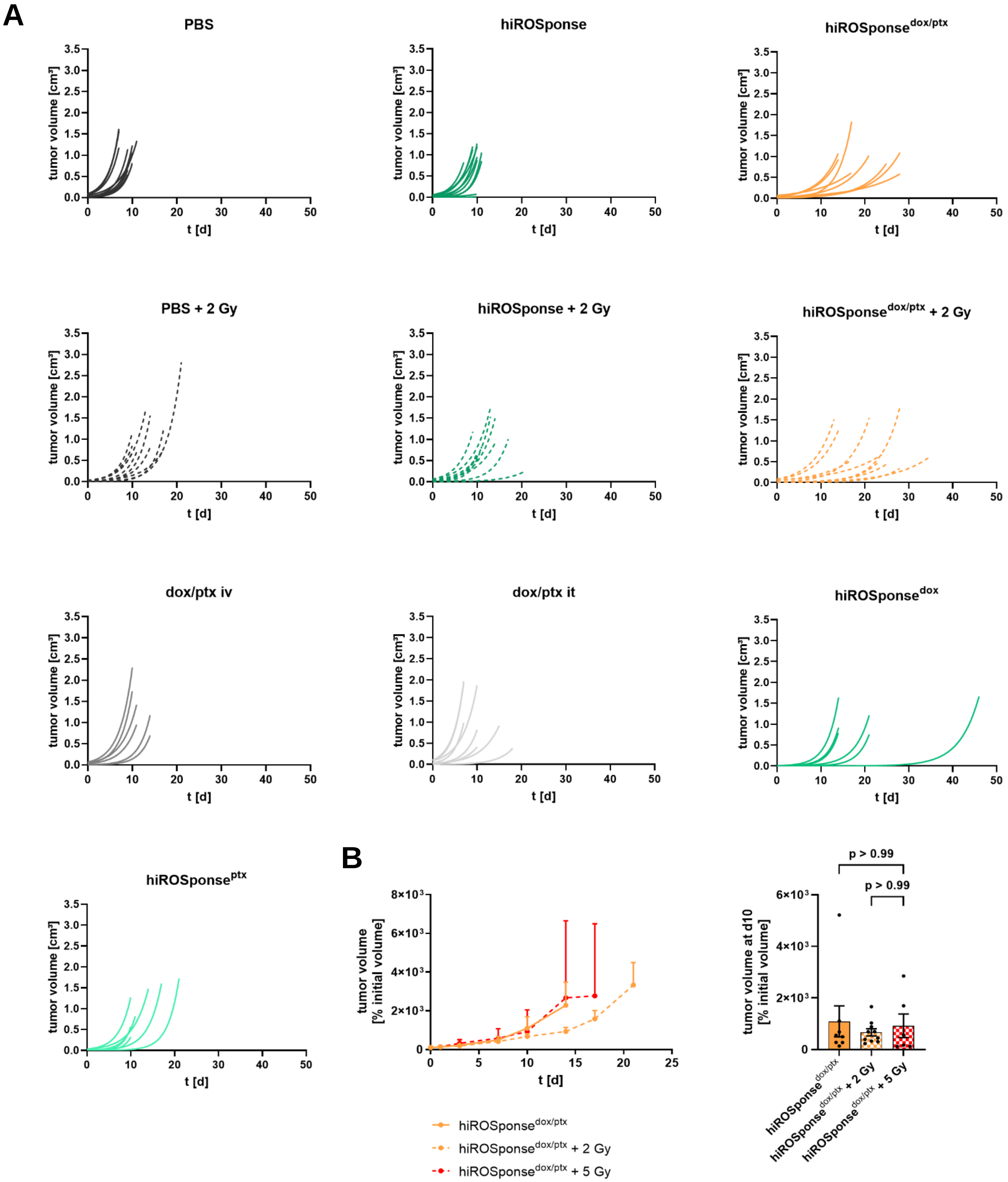

Figure S4A. Exponential growth of melanotic B16F10 tumors in C57BL/6JRj mice modeled with GraphPad Prism software based on quantified tumor volumes from MRI measurements (d0 represents time point of hydrogel injection), n = 6-11. **B.** Comparison of tumor volume after application of hiROSponse$^{dox/ptx}$ without and with external irradiation of 2 Gy or 5 Gy, left: n = 6-11 at d0, mean + SD, right: n = 6-10, mean ± SEM, one-way ANOVA, Bonferroni *post-hoc* test.

Table S2: Calculated parameters of tumor growth and hydrogel degradation in B16F10 melanoma-bearing C57BL/6JRj mice.

group	tumor growth		hydrogel degradation
	**doubling time ± SD [d]**	**rate constant ± SEM [d^{-1}]**	**slope ± SEM [%V/d]**
hiROSponse	1.60 ± 0.40	0.43 ± 0.13	-8.22 ± 0.65
hiROSponse + 2 Gy	2.17 ± 0.47	0.32 ± 0.09	-9.17 ± 0.72
hiROSponse$^{dox/ptx}$	3.34 ± 1.33	0.21 ± 0.08	-5.48 ± 0.46
hiROSponse$^{dox/ptx}$ + 2 Gy	4.21 ± 1.21	0.17 ± 0.03	-4.36 ± 0.29
hiROSponse^{dox}	1.52 ± 0.50	0.40 ± 0.21	-5.46 ± 0.41
hiROSponse^{ptx}	1.50 ± 0.34	0.46 ± 0.18	-4.19 ± 0.46
PBS	1.86 ± 0.25	0.51 ± 0.18	-
PBS + 2 Gy	2.37 ± 0.36	0.29 ± 0.05	-
dox/ptx iv (5 mg/kg)	1.56 ± 0.21	0.45 ± 0.09	-
dox/ptx it (5 mg/kg)	2.36 ± 0.44	0.29 ± 0.06	-

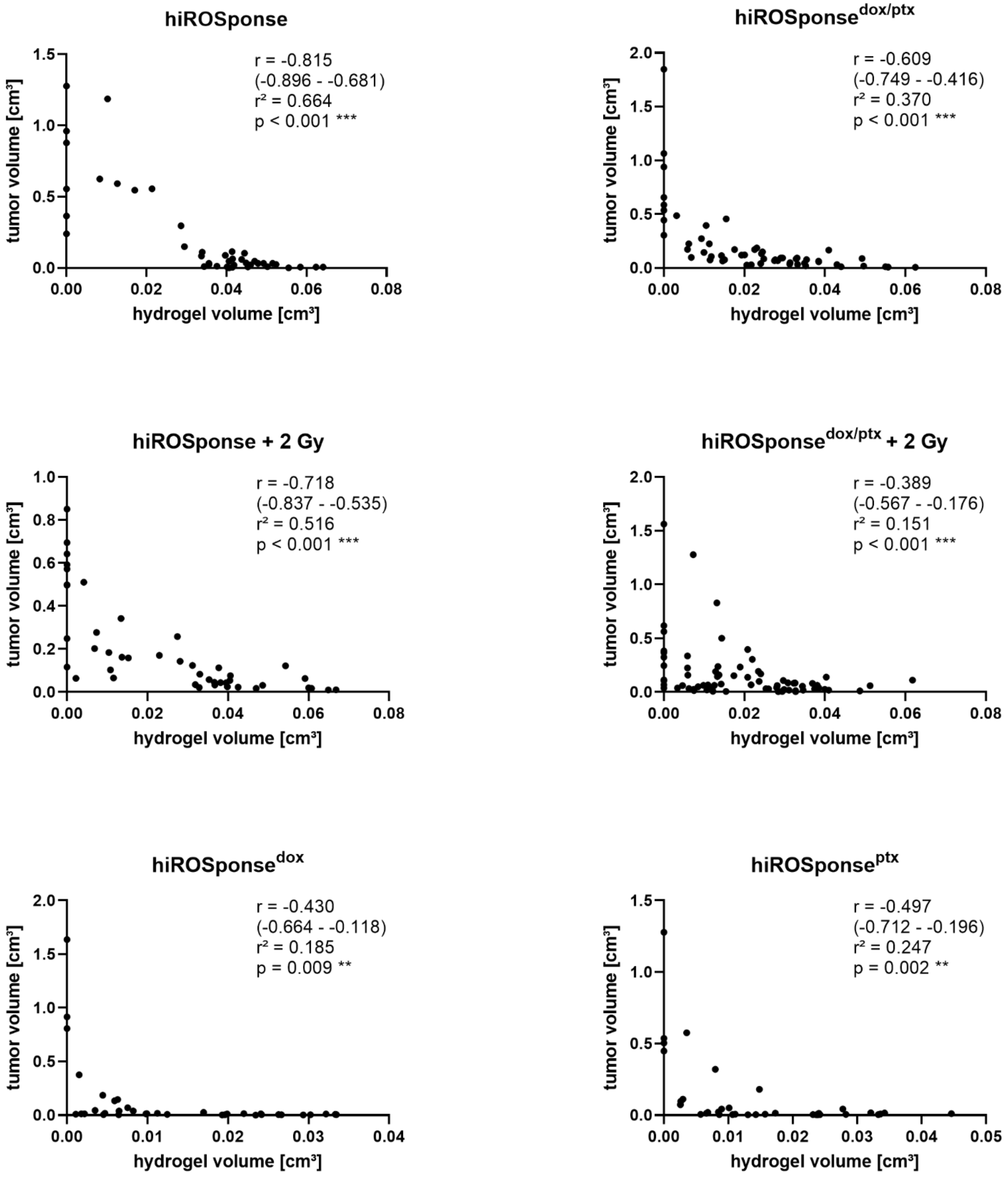

Figure S5: Correlation between B16F10 tumor and hydrogel volume calculated with GraphPad Prism software based on MRI measurements.

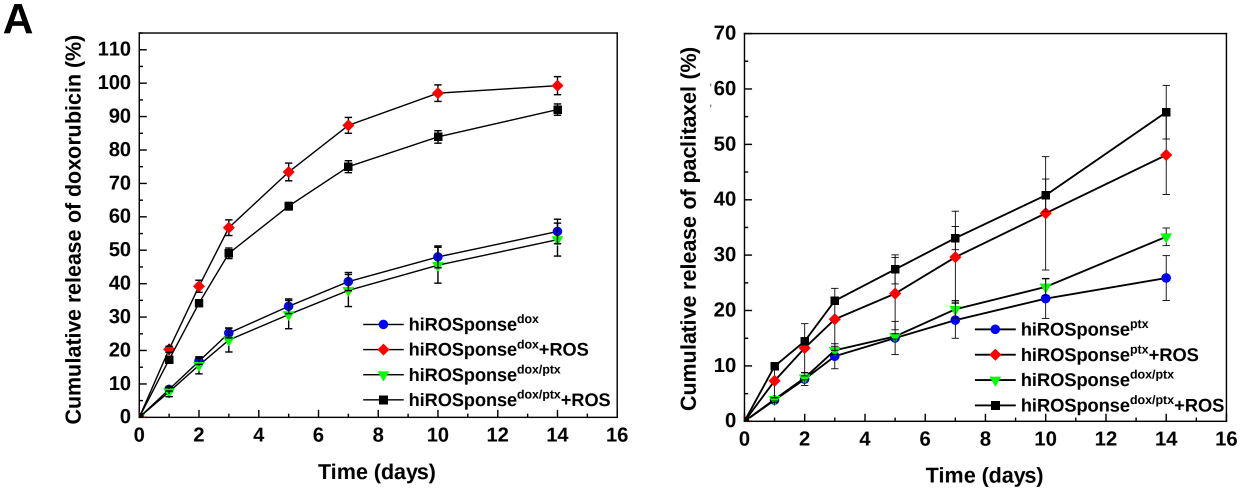

B

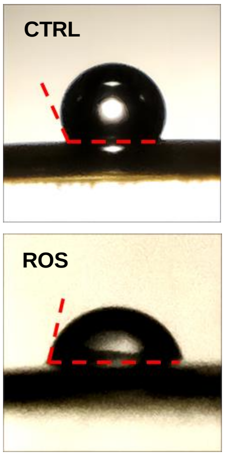

	CTRL	ROS
Sample 1	119. 7°	78.46°
Sample 2	121.06°	75.74°
Sample 3	120.63°	76. 65°

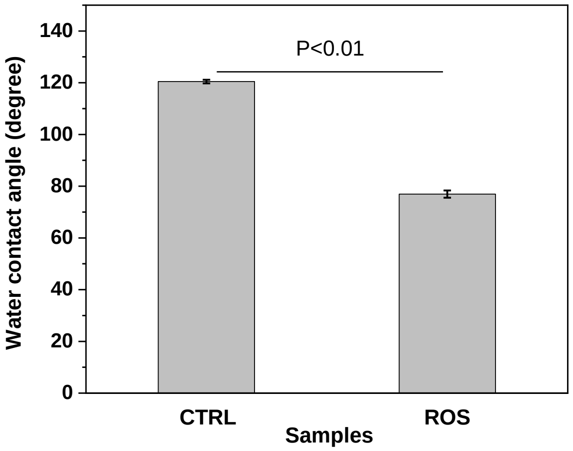

Figure S6. **Drug release and hydrophilicity of hiROSponse in vitro. A.** Cumulative release of doxorubicin (left) and paclitaxel (right) from hiROSponse^{dox}, hiROSponse^{ptx} and hiROSponse$^{dox/ptx}$ in the presence and absence of ROS. **B.** Water contact angle test of hiROSponse in the absence (CTRL) and presence of ROS, n = 3, mean ± SEM, one-way ANOVA, Bonferroni *post-hoc* test.

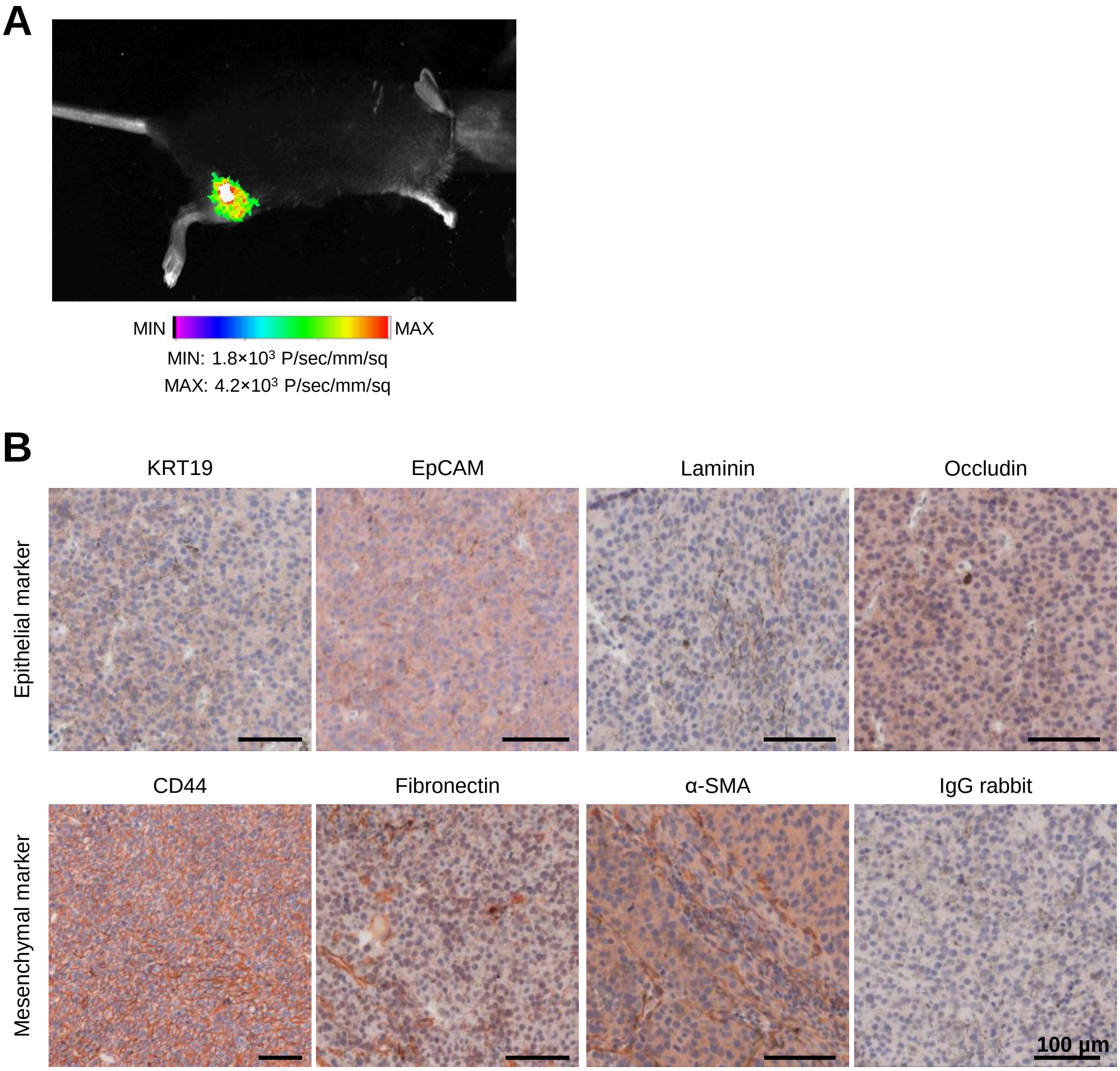

Figure S7A. Representative in vivo chemiluminescence imaging of ROS in B16F10 tumor microenvironment using luminol derivative L-012 with an exposure time of 5 min is shown. **B.** B16F10 tumors immunohistochemically stained for epithelial (cytokeratin-19, epithelial cell adhesion molecule, laminin, and occludin) and mesenchymal markers (hyaluronic acid receptor CD44, fibronectin, and alpha smooth muscle actin) with positive protein staining in red and nuclei stained in blue

A

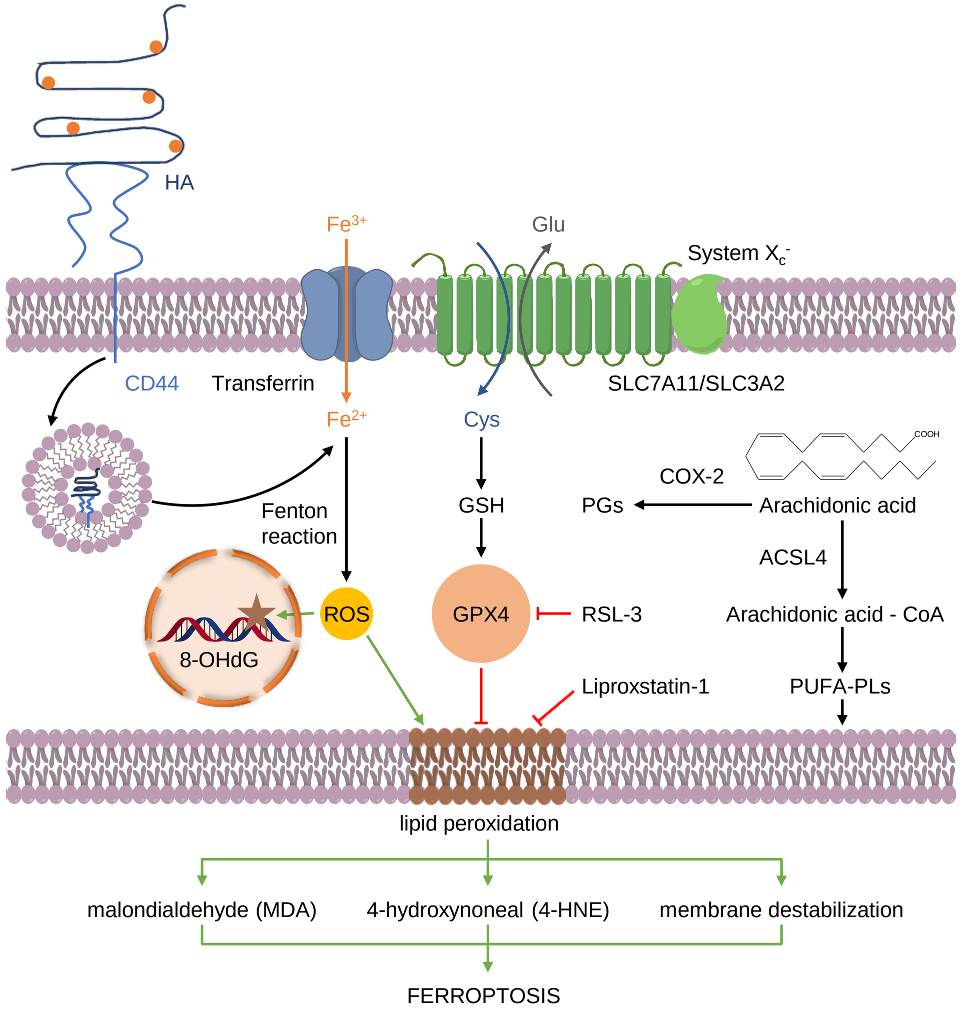

B

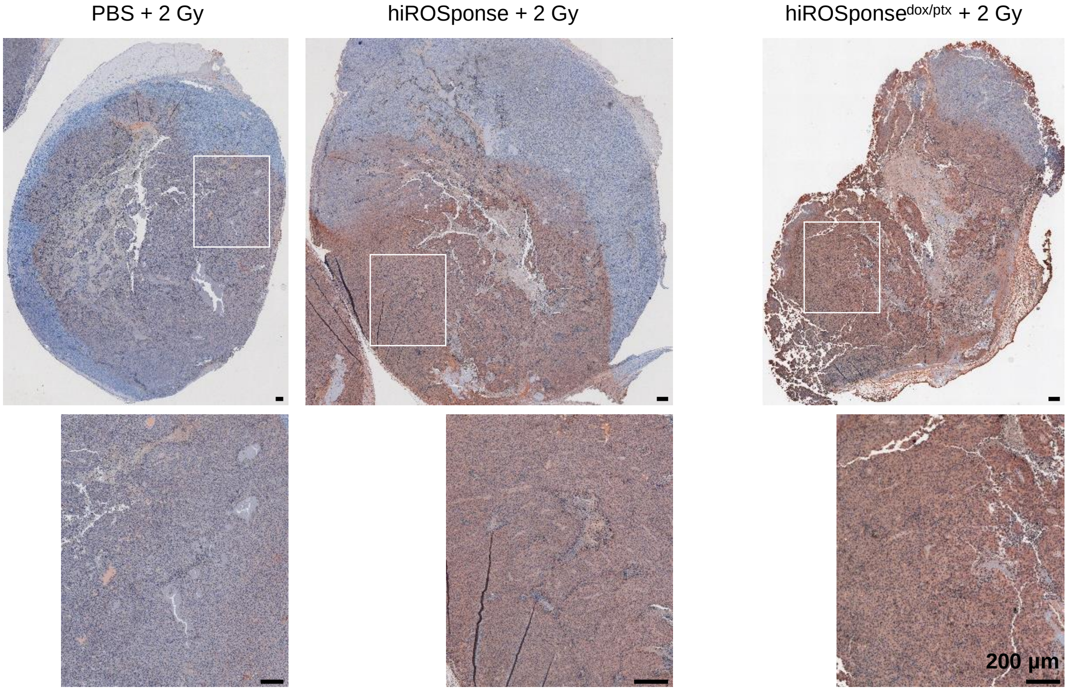

Figure S8A. Scheme of iron- and ROS-dependent lipid peroxidation leading to ferroptosis [according to B. Lu, X. B. Chen, M. D. Ying, Q. J. He, J. Cao, B. Yang, *Frontiers in Pharmacology* **2018**, 8, https://doi.org/10.3389/fphar.2017.00992]. GSH: glutathione, GPX4: glutathione peroxidase 4, RSL-3: RAS-selective lethal (inhibitor of GPX4, inducer of ferroptosis), SLC7A11/SLC3A2: solute carrier family 7 member 11/solute carrier family 3 member 2, COX-2: cyclooxygenase-2 PGs: prostaglandins, ACSL4: acyl-CoA synthetase long chain family member 4, PUFA-PLs: polyunsaturated fatty acids-phospholipids. **B.** 4-hydroxynonenal indicating lipid peroxidation in B16F10 tumors. Depicted are exemplary images of immunohistochemical 4-HNE staining using anti-4-HNE antiserum (HNE11-S, Alpha Diagnostic Intl. Inc., 1:500, AB_2629282).

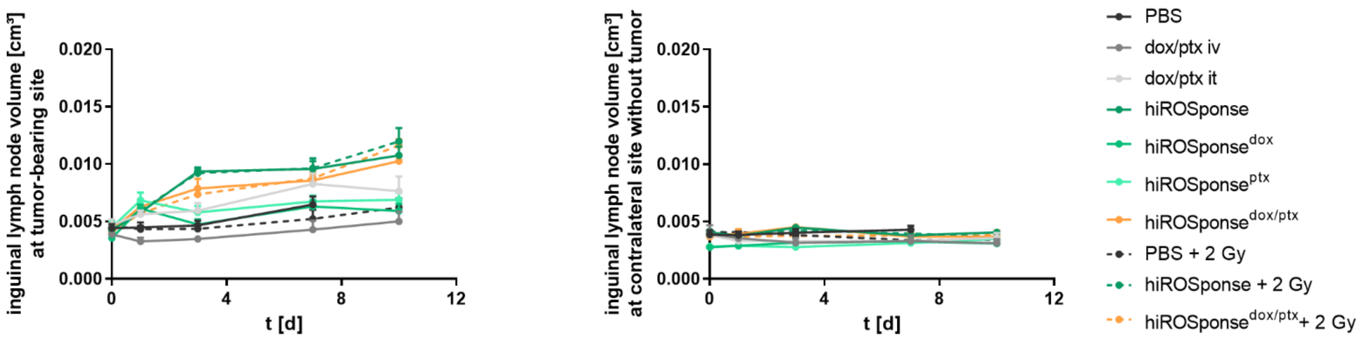

Figure S9: **Inguinal lymph node size of B16F10 melanoma-bearing C57BL/6JRj mice.** Quantified volumes of inguinal lymph nodes at tumor-bearing site (left) and contralateral site without B16F10 tumor (right); n = 6-11, mean + SEM.

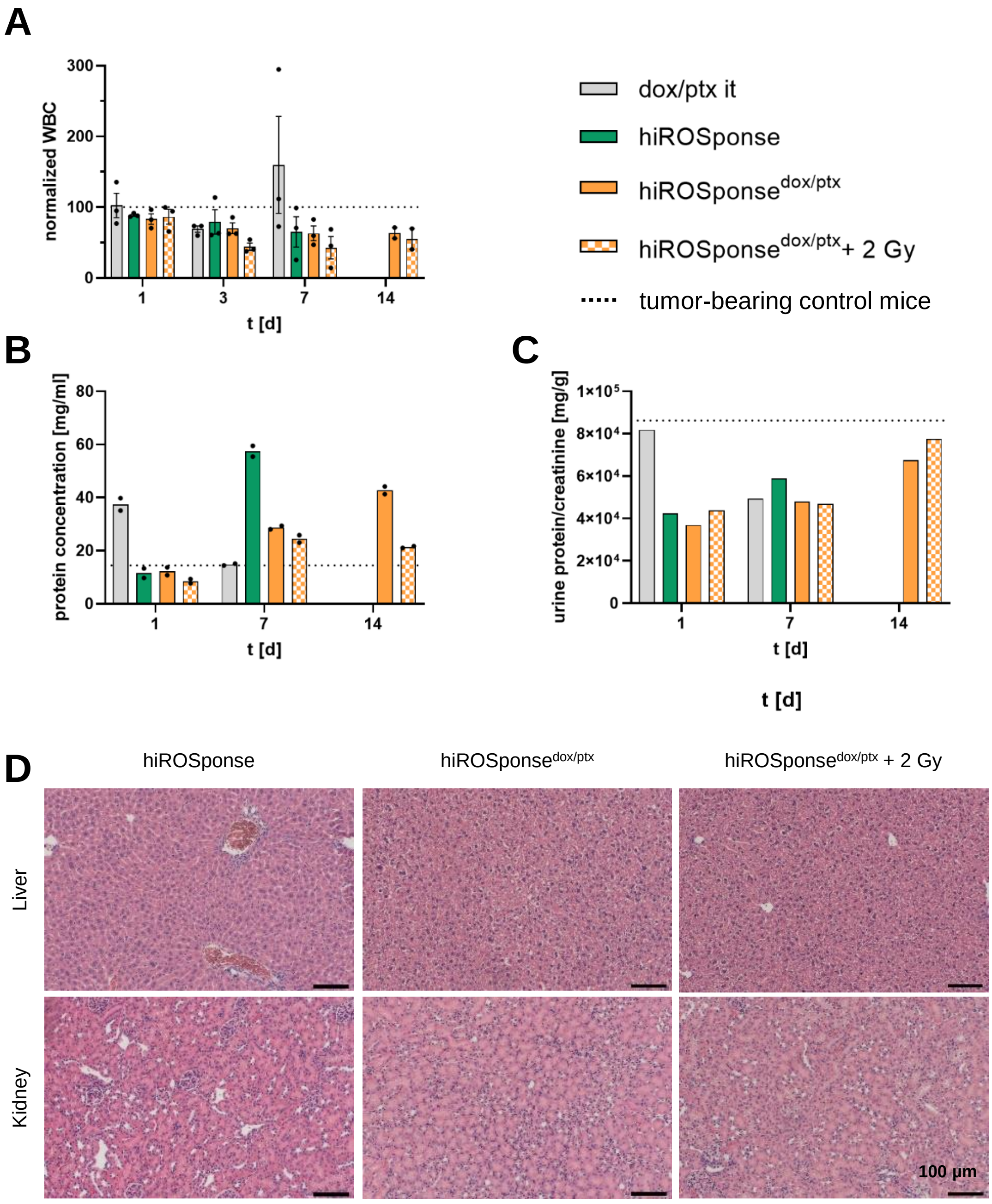

Figure S10: **Analysis of systemic side effects regarding white blood cell count, proteinuria, and morphological alterations in excretory organs in B16F10 melanoma-bearing C57BL/6JRj mice. A.** White blood cell counts normalized to B16F10 melanoma-bearing C57BL/6JRj mice without hydrogel injection; n = 3, mean ± SEM. **B.** Protein concentration in spontaneous urine samples taken at certain time points; pooled samples of three mice per group analyzed in duplicates. **C.** Mouse urine protein to creatinine ratio; pooled samples of three mice per group. **D.** Representative images of H&E stained end-point liver and kidney samples.

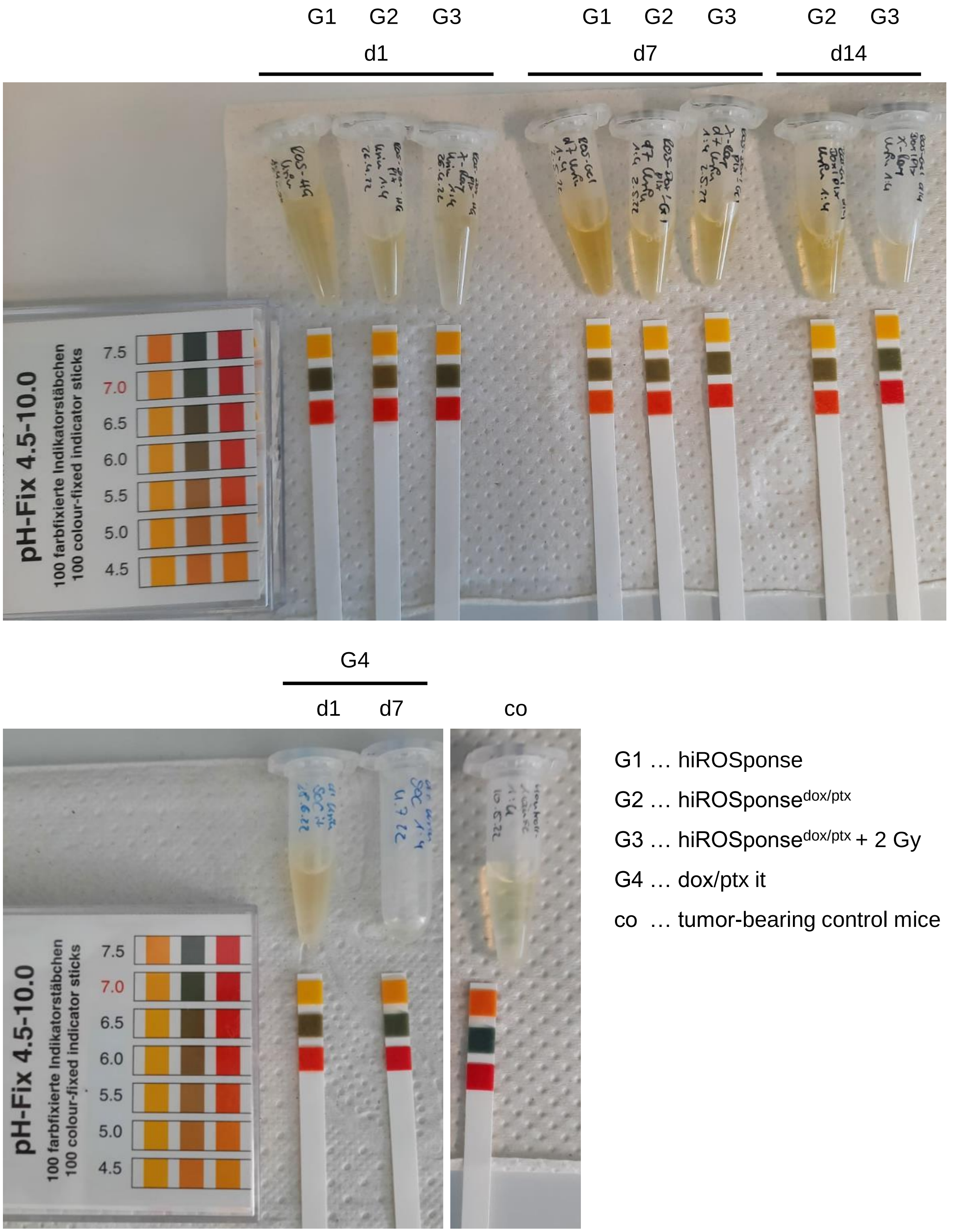

Figure S11: **Spontaneous mouse urine collected from B16F10 melanoma-bearing C57BL/6JRj mice.** Determination of the pH value of pooled spontaneous mouse urine samples at certain days by pH indicator sticks.

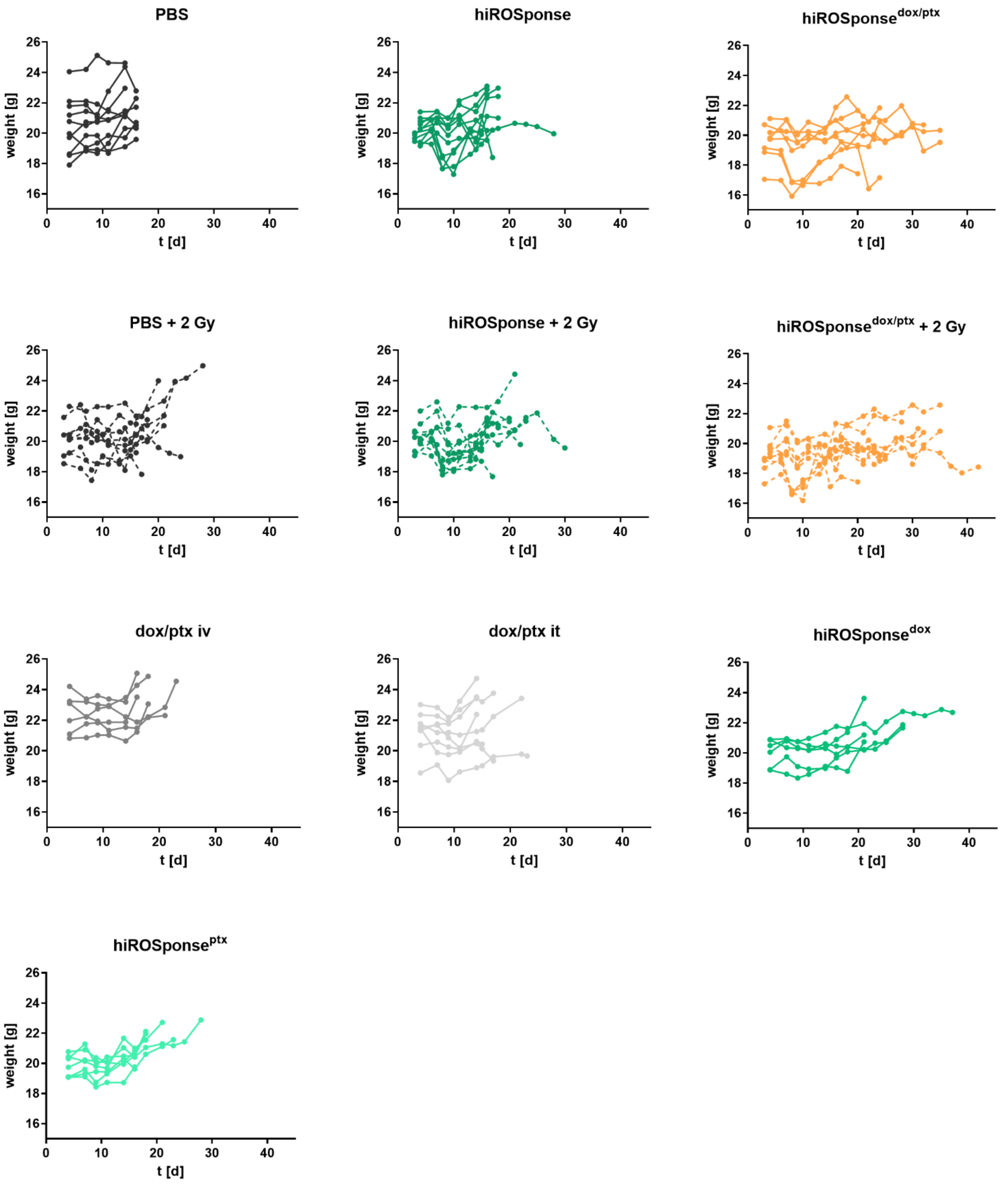

Figure S12: Weight of B16F10 melanoma-bearing C57BL/6JRj mice (d0 represents time point of tumor cell injection), n = 6-11.

Table S3: **Cell line authentication by ATCC.** The table includes the STR analysis of B16F0, F16F1, B16F10 and B78H1 cells for 18 loci. The equation given is used to calculate the percentage match.

**Locus**	**B16F0**	**B16F1**	**B16F10**	**B78H1**
18-3	15, 16	15, 16	15, 16	14, 16
4-2	20.3, 21.3	20.3, 21.3	20.3, 21.3	20.3, 21.3
6-7	15	15	15	15
19-2	13	13	13, 14	13
1-2	19, 20	19, 20	19, 20	19, 20
7-1	26.2	26.2	26.2	26.2, 27.2
1-1	17, 18	17, 18	17, 18, 19	17
3-2	14, 15	14, 15	14, 15	14
8-1	16, 17	16, 17	16, 17	16
2-1	16	16	16	16
15-3	22.3, 23.3	22.3, 23.3	22.3, 23.3	22.3
6-4	18, 19, 20	18, 19	18, 19	18, 19, 20
11-2	16, 17	16, 17	16, 17	16
17-2	15, 16, 17, 18	15, 16, 17	15, 16, 17	15, 16
12-1	17, 18	17, 18	17, 18	17, 18
5-5	16, 20	16, 20	16, 20	15, 16, 19
X-1	28	28	28	28, 29
13-1	17, 18	17, 18	17, 18	17, 18
**Alleles**				
total	34	32	34	30

$$\%\ \text{match} = \frac{2\ (\text{number of alleles matching})}{(\text{number of query alleles} + \text{number of reference alleles})}$$

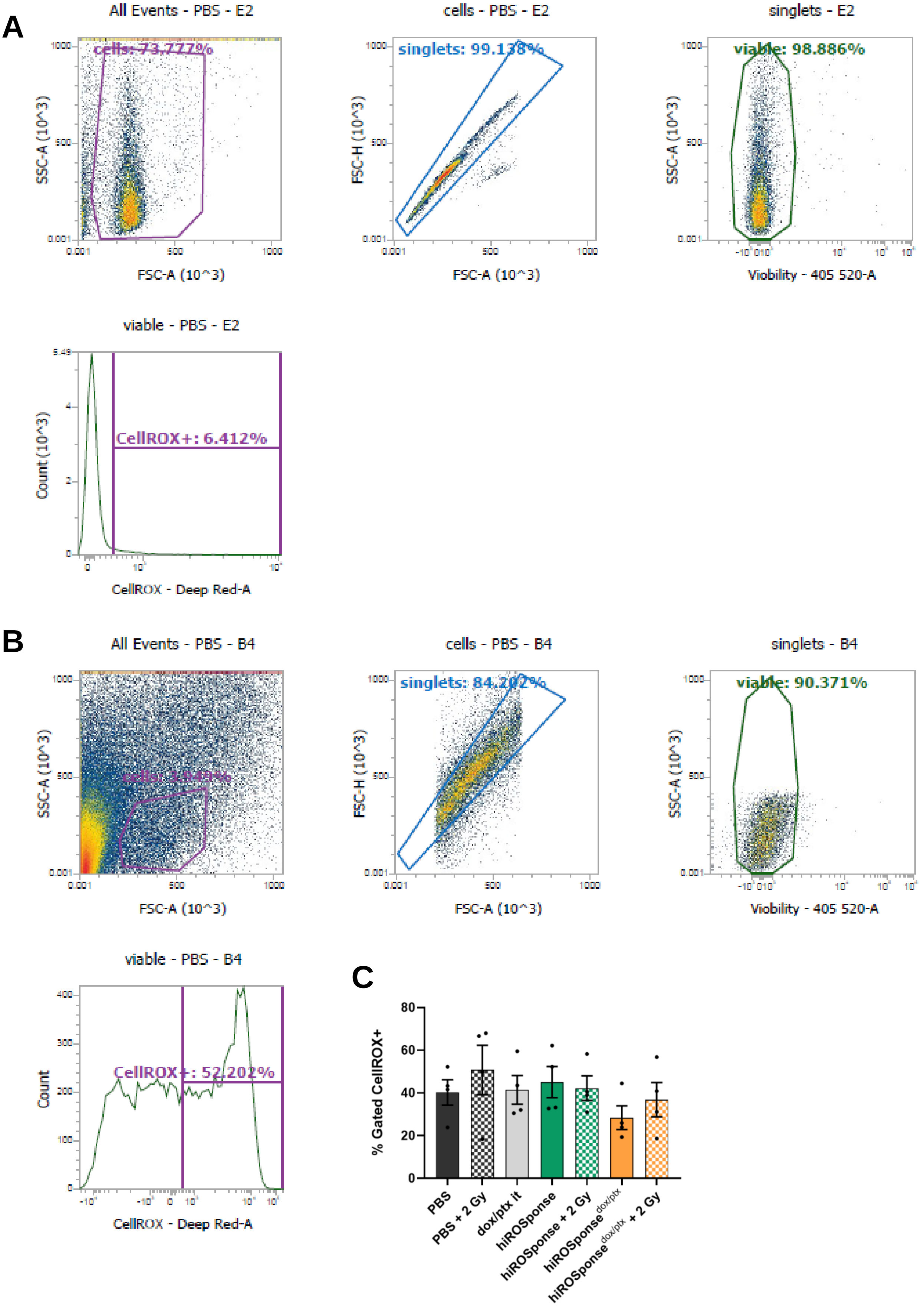

Figure S13: **Determination of ROS level in tumor samples by flow cytometry.** Representative gating strategy of CellROX^{TM} fluorescence signal in **A.** resected B16F10 tumor samples and **B.** resected B78H1 tumor samples. **C.** Quantification of ROS level in B78H1 tumor samples using flow cytometry by gating cells according to a positive fluorescence signal of the CellROX^{TM} probe, n = 4-7, mean ± SEM.

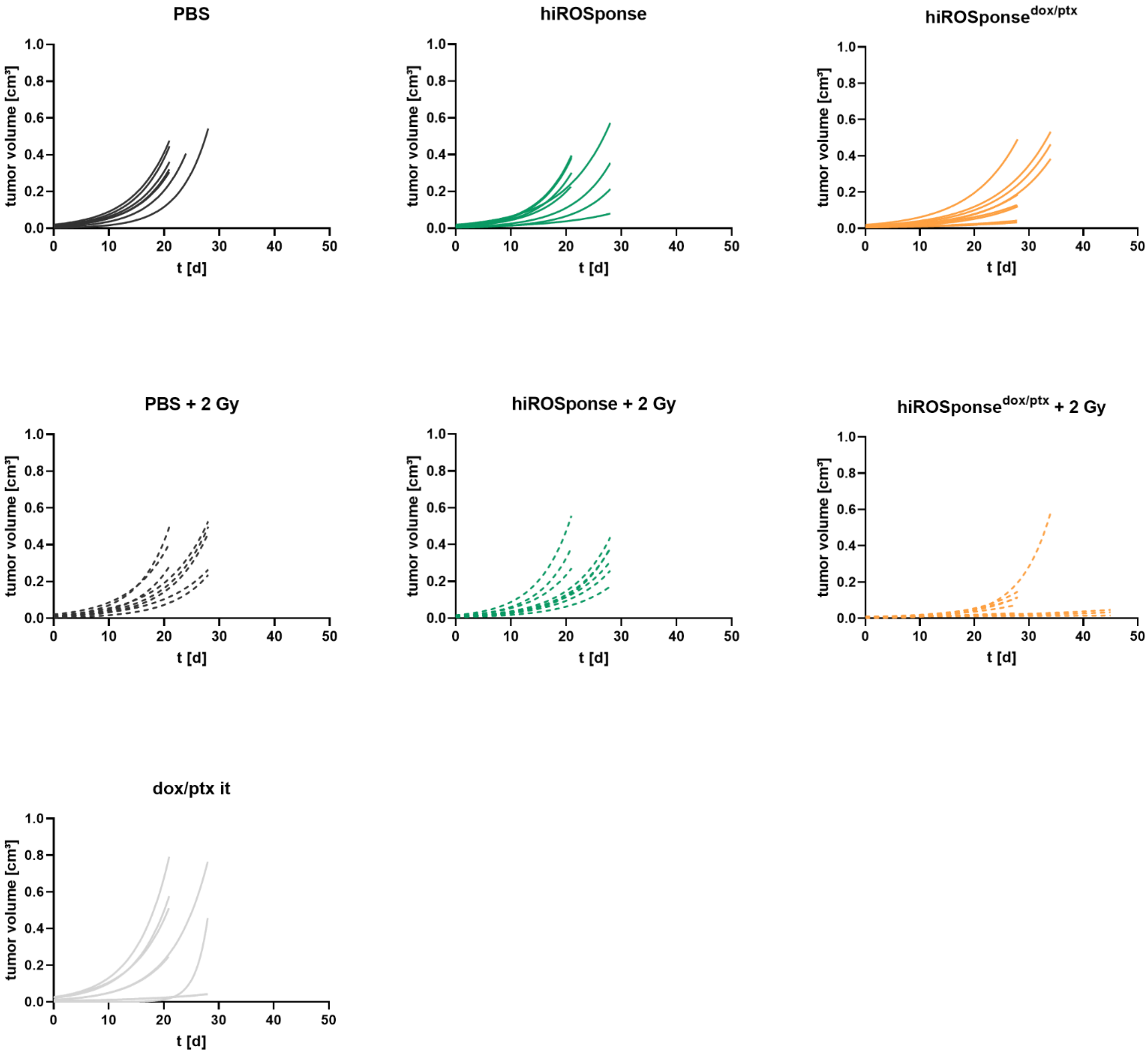

Figure S14. Exponential growth of B78H1 amelanotic tumors in C57BL/6JRj mice modeled with GraphPad Prism software based on quantified tumor volumes from MRI measurements (d0 represents time point of hydrogel injection), n = 7-9.

Table S4: Calculated parameters of tumor growth and hydrogel degradation in B78H1 melanoma-bearing C57BL/6JRj mice.

group	tumor growth		hydrogel degradation
	**doubling time ± SD [d]**	**rate constant ± SEM [d^{-1}]**	**slope ± SEM [%V/d]**
hiROSponse	5.98 ± 1.08	0.12 ± 0.01	-4.16 ± 0.38
hiROSponse + 2 Gy	5.73 ± 0.53	0.12 ± 0.01	-3.95 ± 0.47
hiROSponse$^{dox/ptx}$	6.36 ± 1.53	0.11 ± 0.02	-3.40 ± 0.36
hiROSponse$^{dox/ptx}$ + 2 Gy	16.14 ± 5.57	0.10 ± 0.02	-3.65 ± 0.30
PBS	4.12 ± 0.30	0.17 ± 0.00	-
PBS + 2 Gy	5.51 ± 0.61	0.13 ± 0.01	-
dox/ptx it (5 mg/kg)	5.66 ± 2.43	0.12 ± 0.02	-

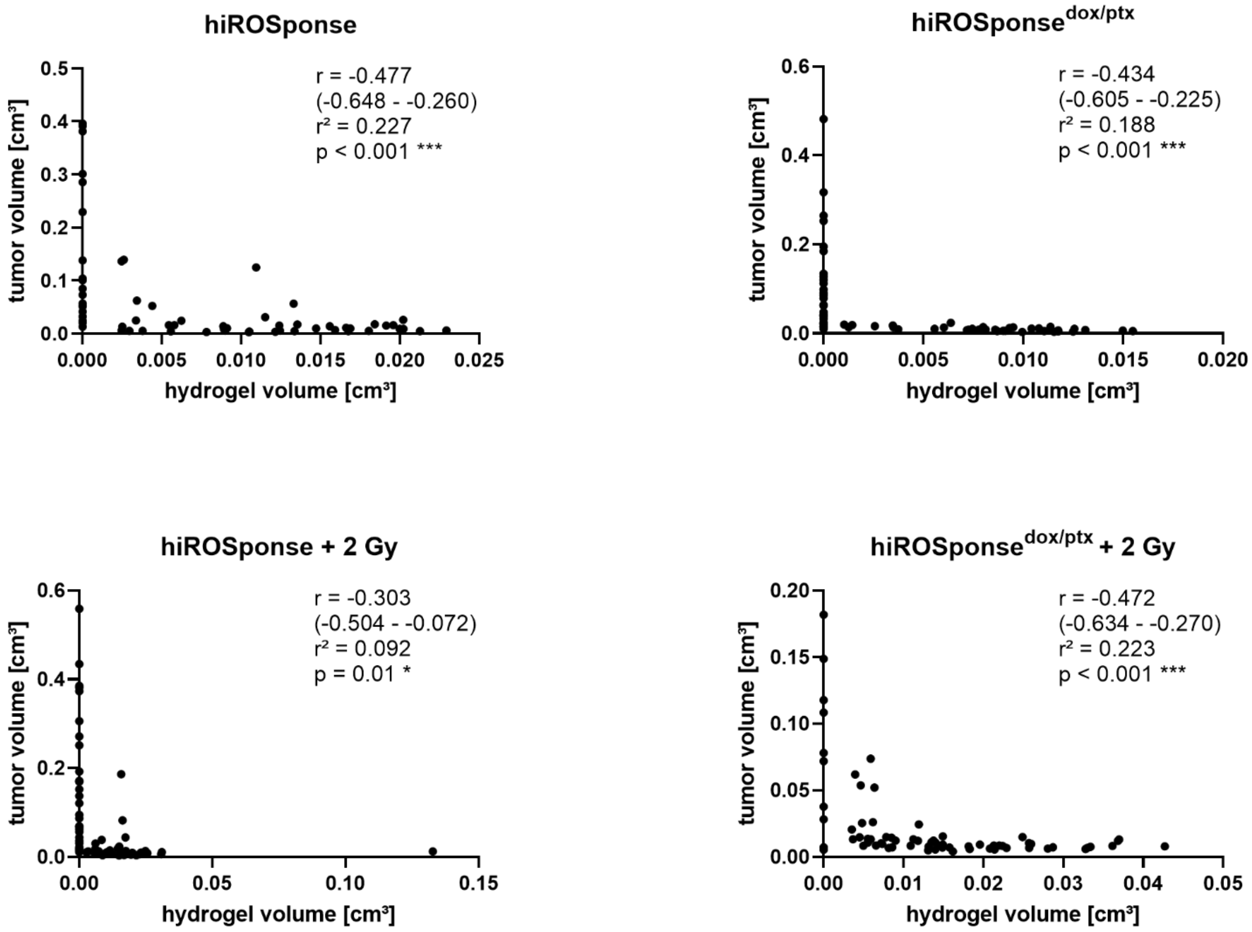

Figure S15: Correlation between B78H1 tumor and hydrogel volume calculated with GraphPad Prism software based on MRI measurements.

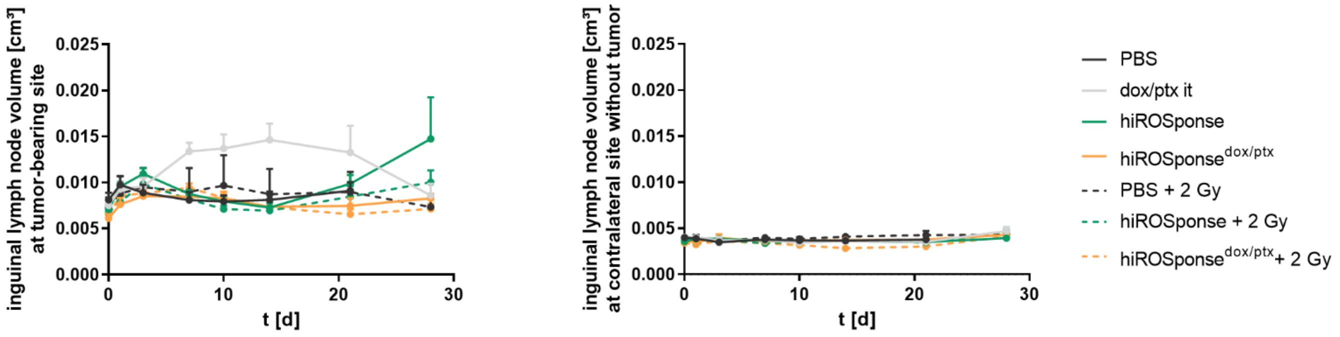

Figure S16: **Inguinal lymph node size of B78H1 melanoma-bearing C57BL/6JRj mice.** Quantified volumes of inguinal lymph nodes at tumor-bearing site (left) and contralateral site without amelanotic B78H1 tumor (right); n = 7-9, mean + SEM.

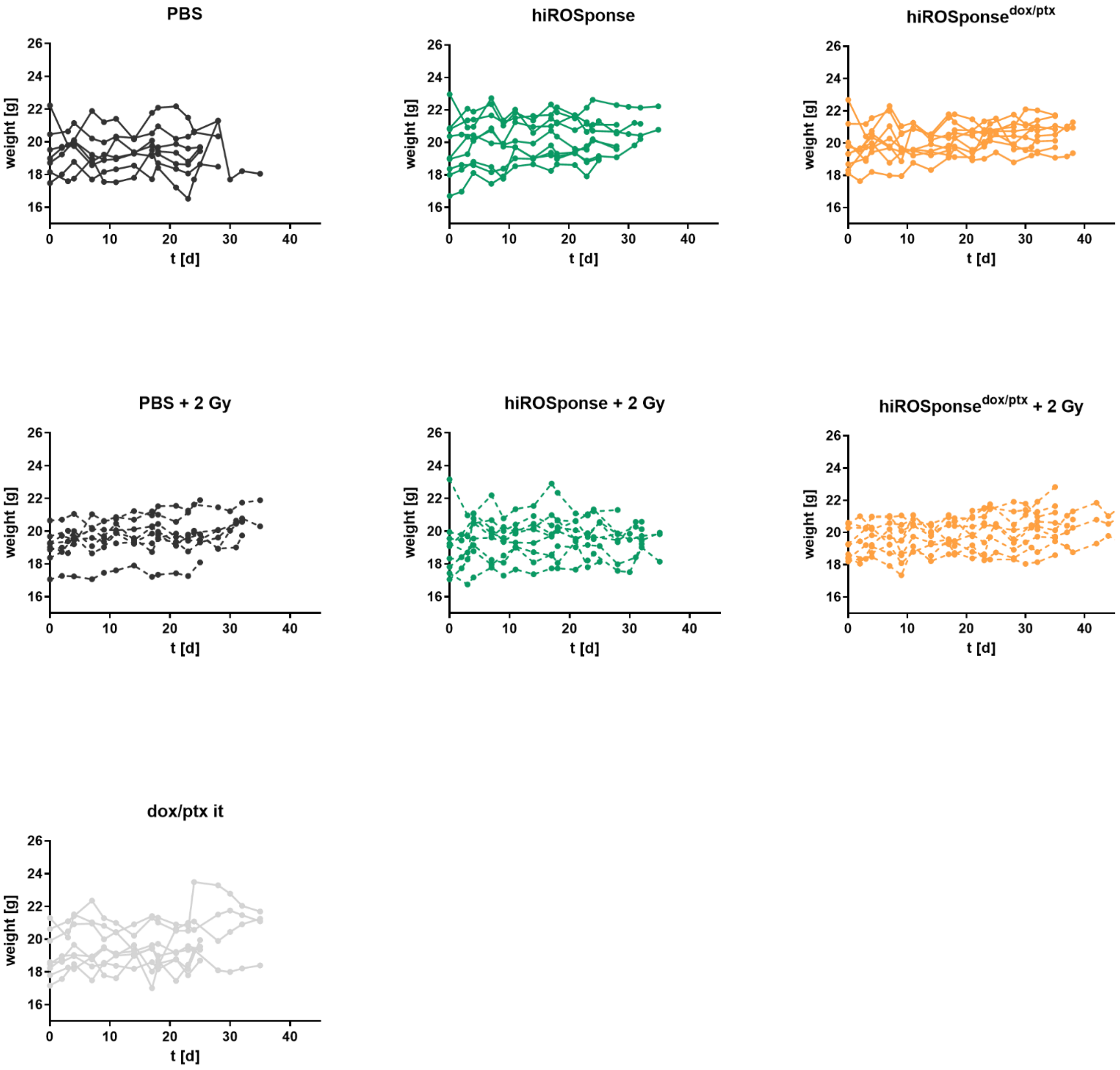

Figure S17: Weight of B78H1 melanoma-bearing C57BL/6JRj mice (d0 represents time point of tumor cell injection), n = 7-9.

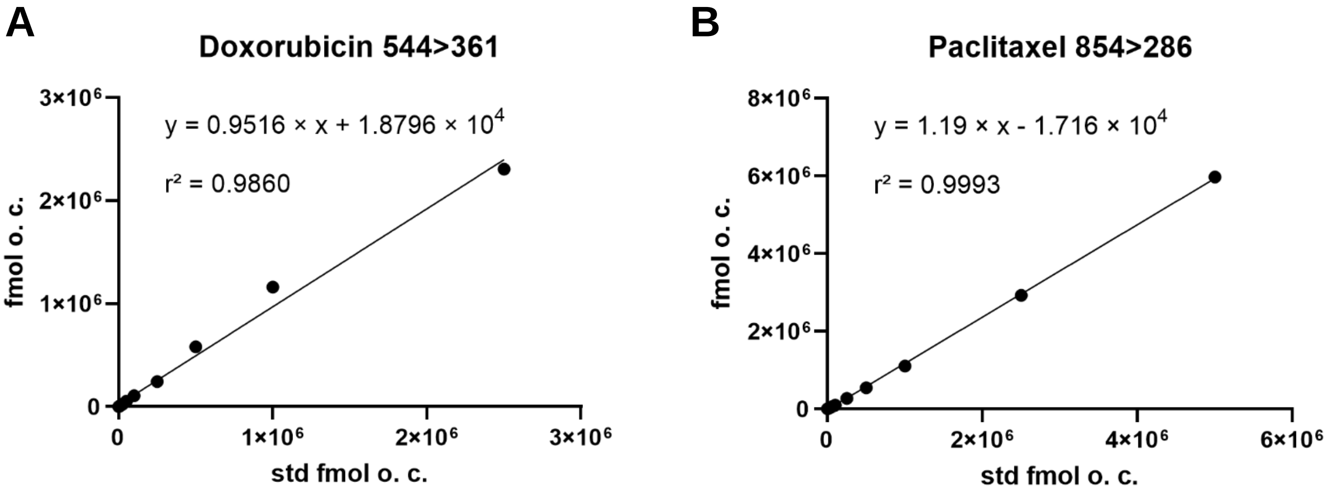

Figure S18: **Calibration of dox and ptx concentration measurements in blood plasma samples by mass spectrometry.** Calibration curves for **A.** dox (50 pM – 500 nM; 2 µl and 5µl injection volume; 544-361) and **B.** ptx (50 pM – 500 nM; 2 µl, 5µl, and 10 µl injection volume; 854-286).

A

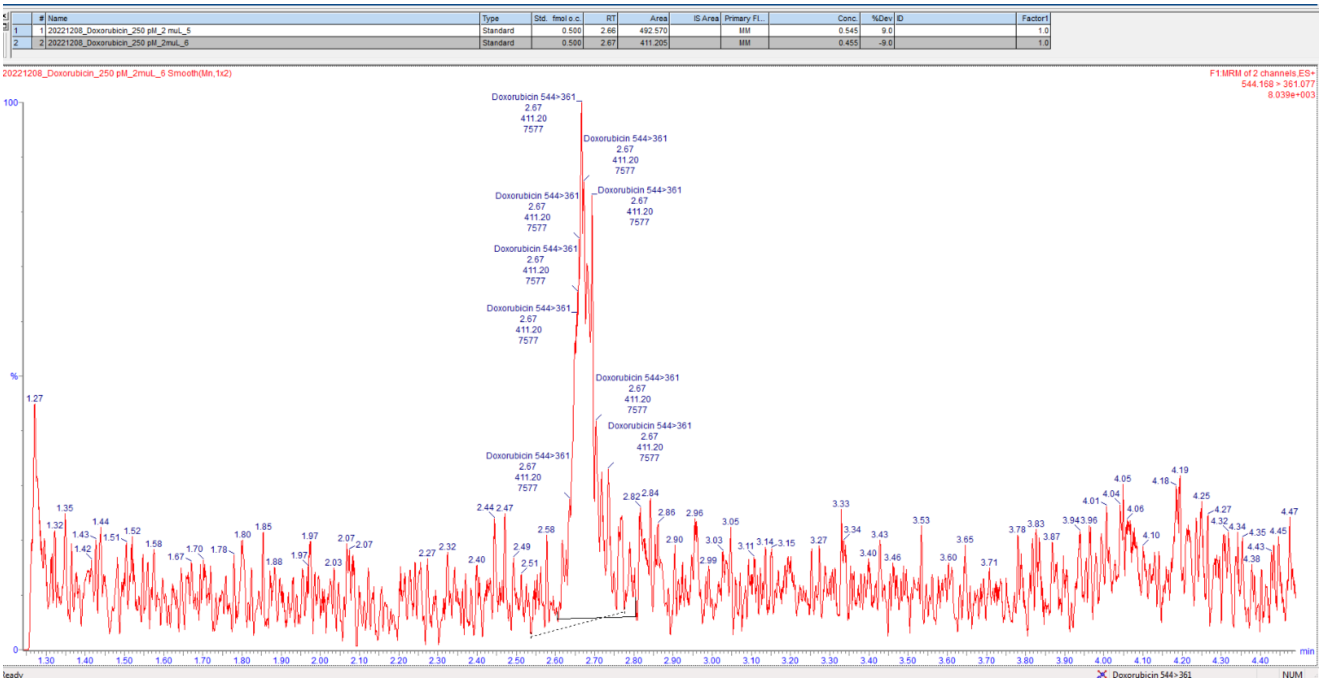

B

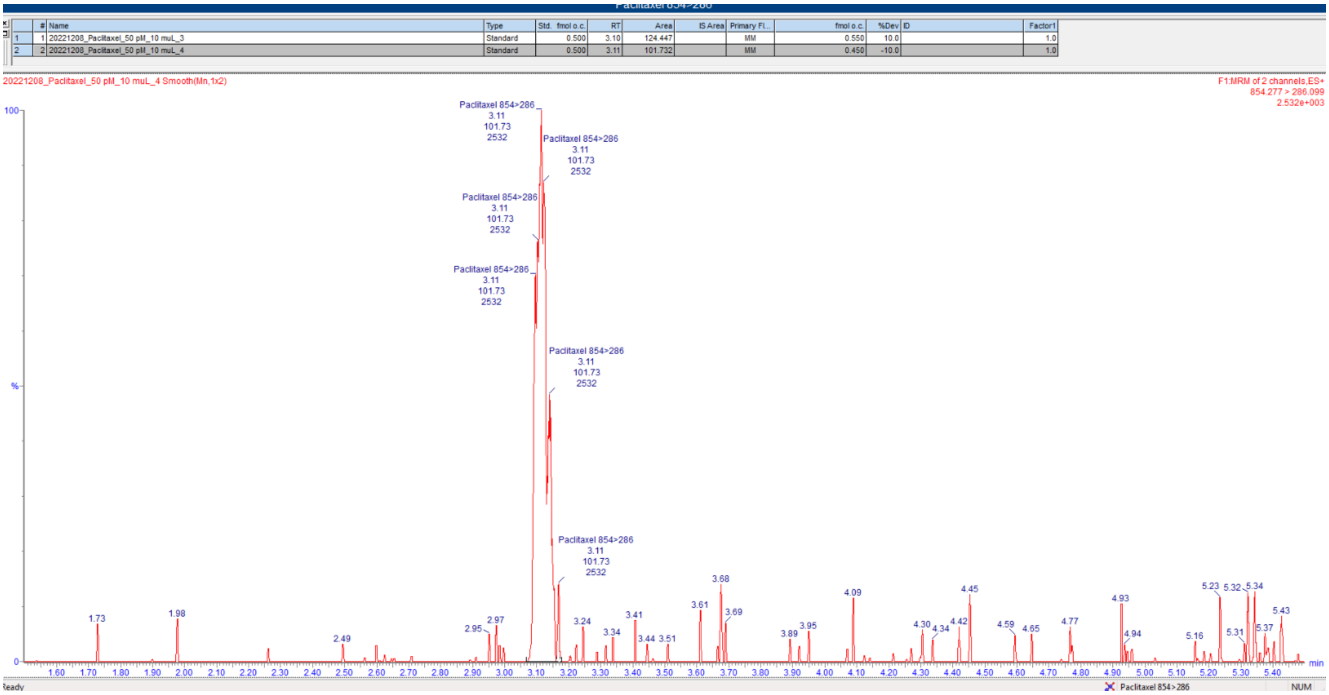

Figure S19: **Determination of dox and ptx in blood plasma samples by mass spectrometry.** Representative chromatogram of **A.** dox measurement (0.5 fmol o.c.; 544-361) and **B.** ptx measurement (0.5 fmol o.c.; 854-286).

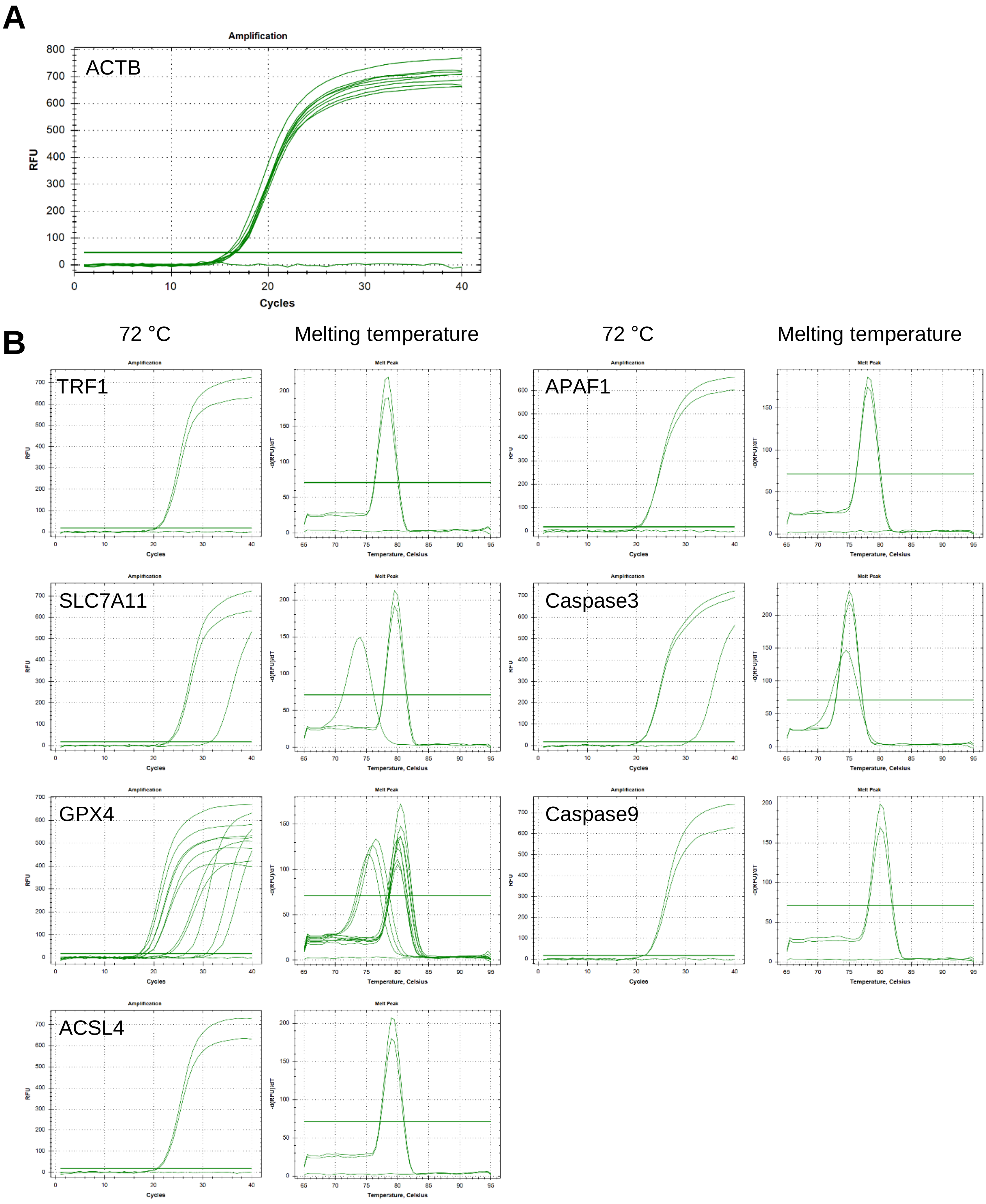

Figure S20. Annealing and melting curves of primers for **A.** housekeeping gene ACTB (beta-actin) being the most stable housekeeping gene among all tested housekeeping genes of the kit and **B.** gene-specific primers for ferroptosis (TRF1, SLC7A11, GPX4, ACSL4) and apoptosis markers (APAF1, Caspase3, Caspase9).
